# 2D and 3D Nanostructured Metal Oxide Composites as Promising Materials for Electrochemical Energy Storage Techniques: Synthesis Methods and Properties

**DOI:** 10.3390/ijms252312521

**Published:** 2024-11-21

**Authors:** Cornelia Bandas, Corina Orha, Mircea Nicolaescu, Mina-Ionela Morariu (Popescu), Carmen Lăzău

**Affiliations:** 1 Condensed Matter Department, National Institute for Research and Development in Electrochemistry and Condensed Matter, 300224 Timisoara, Romania; cornelia.bandas@gmail.com (C.B.); orha.corina@gmail.com (C.O.); mircea.nicolaescu@student.upt.ro (M.N.); mina.popescu@student.upt.ro (M.-I.M.); 2 Department of Applied Chemistry and Engineering of Inorganic Compounds and Environment, Politehnica University of Timisoara, 300223 Timisoara, Romania

**Keywords:** supercapacitor, metal oxide, 2D structure, 3D structure, composite materials

## Abstract

Due to population growth and global technological development, energy consumption has increased exponentially. The global energy crisis opens up many hotly debated topics regarding energy generation and consumption. Not only is energy production in short supply due to limited energy resources but efficient and sustainable storage has become a very important goal. Currently, there are energy storage devices such as batteries, capacitors, and super-capacitors. Supercapacitors or electrochemical capacitors can be very advantageous replacements for batteries and capacitors because they can achieve higher power density and energy density characteristics. The evolution and progress of society demand the use of innovative and composite nanostructured metal oxide materials, which fulfill the requirements of high-performance technologies. This review mainly addresses the synthesis techniques and properties of 2D and 3D metal oxide nanostructured materials, especially based on Ti, Fe, Ga, and Sn ions, electrochemical methods used for the characterization and application of 2D, and 3D nanostructured metal oxide structures in electrochemical storage systems of energy.

## 1. Introduction

As energy consumption becomes more common in every aspect of our lives, there is an increasing requirement for energy storage systems to support renewable energy sources, and conversion technologies are essential for the global scientific and technological communities. In this way, the world of science is focusing on energy because the global landscape is constantly changing. Over time, different technologies have been employed to produce unique energy storage devices such as supercapacitors, batteries, fuel cells, and water-splitting to fulfill the need for hybrid and electric cars, aircraft, electronics, and different types of supplies, among other things [1,2,3,4,5,6]. Furthermore, with the increased deployment of renewable energy sources such as solar power, the need for efficient energy storage solutions is essential to address the duck curve phenomenon. The duck curve represents the fluctuation of grid electricity demand, particularly on days when solar energy production is high and grid demand is low [7]. While direct electrification methods and batteries are able to handle day-to-day fluctuations in solar PV production and demand, longer-term fluctuations (days, weeks, or even months) require high-capacity storage solutions along with low energy costs. For long periods of time, chemical storage systems are perfectly suited. Due to their advantages such as low cost and long lifespan, supercapacitors have also emerged as promising storage methods for balancing energy needs [8].

**Figure 1:**
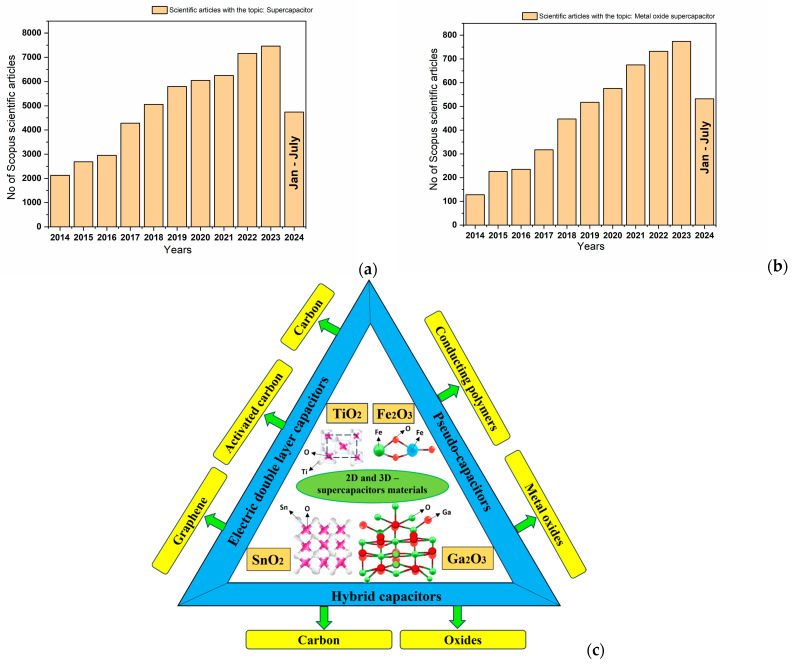
A rise in the number between 2014 and 2024 [Source Scopus date 1 August 2024] of supercapacitors publications (**a**) and of metal oxide supercapacitors (**b**); schematic diagram of the review content (**c**).

Batteries and supercapacitors are unconventional power sources used in electric vehicles and portable electronic devices, which are able to convert chemical energy into electrical energy. The US Department of Energy (DOE) has spotlighted batteries and supercapacitors as major future energy storage technologies [9]. Supercapacitors (SCs) are attracting the attention of scientists because of their extraordinary electrochemical characteristics, including higher particular power value, longer life cycle, and high-speed charging and discharging. In the last decade, the interest of scientists has been growing, with research being increasingly focused on the supercapacitors field. Figure 1a shows a list of publications produced in the last decade, illustrating the growth trend in supercapacitor research. Starting from the 1950s, when scientists started research with capacitors based on carbon electrodes, and continuing until 1957 when Becker H. created a low-voltage electrolytic capacitor with a remarkable capacity of energy storage, the field of supercapacitors has seen an extraordinary evolution highlighting several notable results in energy storage. Later, in 1971, the term “pseudocapacitor,” also known also as redox capacitor, appeared. Pseudocapacitors have the ability to store energy by a reversible faradic interaction on the surface of the active elements. The pseudocapacitor charge storage mechanism is critical and controlled by the faradaic charge transfer process [10] that occurs on the electrode’s surface or a few neighboring surface layers. In 1989, a positive electrode and a negative electrode based on activated carbon were developed to form an asymmetric supercapacitor (ASC). After a few years, in 1992, the “Boost Caps” launched. These were an exclusive line of supercapacitors that represented a significant milestone in supercapacitor technology progress [10]. Even if supercapacitors present many advantages, they have a lower specific energy than batteries, thus to overcome this limitation, scientists have focused on developing new electrode materials that might enhance the energy density of supercapacitors. It is well known that transitional and non-transitional metal oxides have electrochemical stability, high specific capacitance, and electrical conductivity, so they are ideal candidates for high-performance supercapacitor electrodes. As illustrated in Figure 1b, interest in metal oxide materials for supercapacitors has increased, with a significant number of articles published in prestigious journals.

In this review, recent developments in 2D and 3D metal oxide nanostructured electrode materials applied as electrochemical energy storage systems (supercapacitor) were investigated together with the impact of electrode structures based on transitional metal oxide and non-transitional metal oxides and their nanocomposites. A comprehensive coverage of the synthesis methods together with the specific characteristics and application area of supercapacitors were also related. A schematic diagram is illustrated in Figure 1c about the overview of the review. Finally, an outlook covering the directions and challenges for the development of advanced energy storage materials is provided.

## 2. Supercapacitors

**Figure 2:**
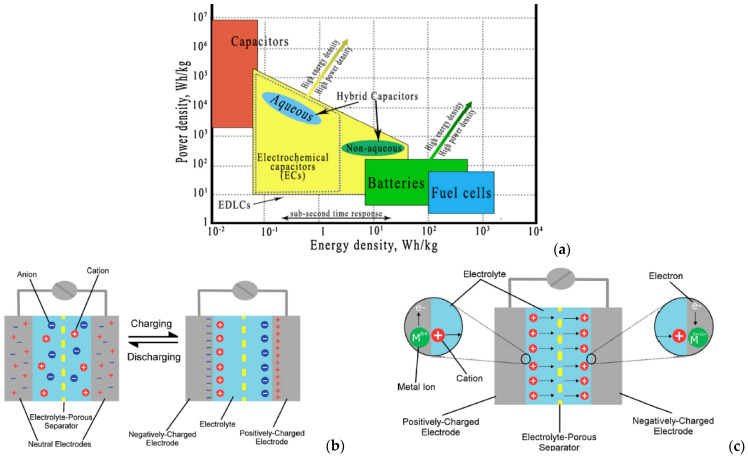
Ragone plot of performance ranges of various energy storage devices. Reproduced with permissions from ref [15], copyright 2019, Elsevier (**a**); EDLC supercapacitor—schematic charging/discharging process (**b**) and Pseudocapacitor—schematic mechanism (**c**); Reproduced with permissions from ref [16], copyright 2018, Elsevier.

Supercapacitors, known as ultracapacitors (commercial name given by Nippon Electric Company in Japan, and by Pinnacle Research Institute, PRI, in the United States) or electrochemical double-layer capacitors (technical name), are devices that can be utilized as energy storage systems, with high energy and power densities, a long lifespan and a high efficiency up to 95% [11,12]. Supercapacitors, with their long cycle life and excellent power delivery, are useful in both commercial and military applications. Thus, Coleman offers supercapacitor-powered cordless screwdrivers for household use, which are fully charged within 90 s for immediate usage [13]. In the military field, supercapacitors are commonly employed as an alternative power source for electronics in armed vehicles and black boxes on helicopters [14]. Supercapacitors store charge similarly to regular capacitors; however, the charge accumulates at the contact between the surface of a conductor and an electrolytic solution rather than in two conductors. Supercapacitor systems include two electrodes that have a potential to be applied across the cell, resulting in two double-layers, one at each electrode/electrolyte interface. To prevent electrical contact, an ion-permeable separator is placed between the electrodes, allowing ions from the electrolyte to pass through (anions to the positive electrode, and cations to the negative electrode). This is the reason that this type of capacitance is also known as Electrochemical Double-Layer Capacitance (EDLC). Figure 2b depicts a schematic of this process. The main feature of a supercapacitor that makes it suitable for use in electrochemical storage systems is the ability to charge and discharge quickly without losing performance over thousands of cycles, as it stores electrical energy directly [15]. A great property of supercapacitor devices is that it recharges in a very short time and can supply frequent and high-power needs.

Since no chemical processes take place and the electrolyte is not depleted in any way, the EDLC mechanism allows SCs to be cycled repeatedly. Another important finding is that there is no volume change in EDLC. This is important since a volume shift would exert stress on the materials and significantly lower the SCs ability to cycle. The energy density attained is still significantly less than that of a battery, even though this performance is far greater than that of a standard capacitor. Pseudocapacitance is frequently used in SCs to reduce some of their cyclability in order to achieve a superior energy density. Energy is stored in the pseudocapacitance through rapid surface redox reactions occurring at the electrode–electrolyte interface. The mechanism is elucidated in Figure 2c.

**Figure 3:**
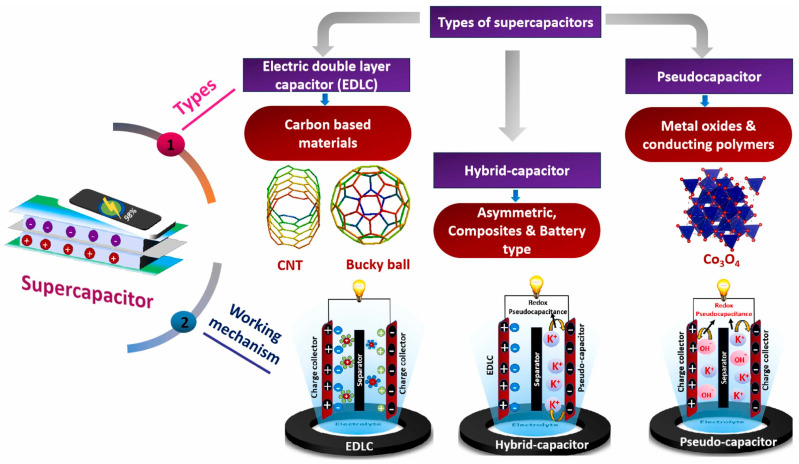
Schematic illustration of the symmetric, asymmetric, and hybrid structures. Reprinted with permission from [18]. Copyright 2024, Elsevier.

The electrochemical reaction in a normal Li-ion battery occurs differently during the charge/discharge phases; so, during the discharge phase, the battery functions as a load, and the working Li electrons move from the anode to the cathode via a separator. Also, the two conducting electrodes of conventional capacitors were separated by an insulating dielectric substance. When the voltage is delivered to the capacitor, opposite charges build up on the surface of each electrode. Figure 2a shows the Ragone plot of the performance ranges of different energy storage systems. By generating an electric field that allows the capacitors to store energy, the dielectric maintains the charge separation, as presented by the schematic in Figure 2b,c. Based on their charge storage mechanism, supercapacitors can be classified into three types: EDLCs, pseudocapacitors, and hybrid supercapacitors, as shown in Figure 3. Amongst the three types of capacitors, EDLCs are considered the most appropriate for the energy storage systems because they offer the most stable charge storage capability for long-term cycling which is the most important criterion for fabricating high performance and stable energy storage systems [17].

### 2.1. Electric Double-Layer Capacitors

The EDLC has been considered a promising high-power energy source for digital communication devices and electric vehicles. Nowadays, EDLCs have attracted significant scientific community attention in the energy storage field as promising energy storage devices because of their features, like their ability to quickly store and deliver a lot of energy compared to batteries and pseudo-capacitors [19]. EDLC, depending on the materials used, can be divided into 3 types: activated carbon, carbon aerogels, and carbon nanotubes/graphene/carbide-derived carbon. Hybrid capacitors can be divided into asymmetric hybrid, rechargeable battery-type, and composite hybrid type, while pseudocapacitors can be divided into two categories: conducting polymers and metal oxides [20]. Different materials such as various carbon based-materials, mixed metal oxides, and hybrid/conducting polymers have been used as electrode materials [21]. Carbon electrodes in their various forms, such as carbon foam, activated carbons, and carbon nanotube/nanofibers, are the most widely used electrodes due to their low cost, availability, and long history of use [22]. In recent years graphene, graphene oxide, or reduced graphene oxide, due to their remarkable characteristics such as chemical stability, high electrical conductivity and large surface area, have also been considered as a promising capacitor electrode materials [23].

The choice of electrolyte has a great impact on the choice of electrode material in an EDLC. There are currently two types of electrolytes in use in EDLCs: organic and aqueous. Thus, when the electrolyte is selected, numerous functional properties that influence the performance of the device are important, such as low viscosity, high ionic conductivity, a wide potential window, low cost, electrochemical stability, and environmentally friendliness [18,24,25]. The most common electrolytes are based on solutions of tetraethylammonium tetrafluoroborate in propylene carbonate or acetonitrile. Among the alternative electrolytes proposed so far, those based on ionic liquids appear to be of particular interest, as their use allows the development of high-energy EDLCs showing operating voltages as high as 3.5 V [26]. The separator, a very important component of SC, prevents the occurrence of electrical contact between the two electrodes, but it is ion-permeable, allowing ionic charge transfer to take place. Synthetic polymer or paper separators can be used with organic electrolytes, and ceramic or glass fiber separators are often used with aqueous electrolytes. In industry, cellulose is often used due to its low cost and good overall performance, whereas the higher costs of synthetic polymers prevent their use in commercial devices. For a higher performance of EDLC, the separator should have thermal and mechanical stability, high electrical resistance, high ionic conductance, low thickness, and low chance of material failure during operation [27,28].

### 2.2. Pseudocapacitors

Another type of supercapacitor is the pseudocapacitor, in which the electrode’s electrochemical effect acts like a capacitor, which results in a linear relationship between the charge storage and the potential window range [29]. Pseudocapacitors combine properties such as the high energy density of batteries with the fast charge/discharge rates and long cycle life of capacitors. Pseudocapacitors are also known as redox supercapacitors or faradaic supercapacitors; this faradaic process leads to pseudocapacitors with higher energy densities than EDLCs. Most electrode materials of this type of capacitor include metal oxides and conductive polymers. However, pseudocapacitors have a shorter life cycle and power density, and these results occur due to the redox reactions in the capacitors [30]. Unlike conventional capacitors and batteries, pseudocapacitors have several properties, such as high energy density, which allows for significantly more energy storage than EDLCs due to the additional charge storage from redox reactions, and fast charge/discharge rates, which allows for high power density, quick response times, and long cycle life, compared to batteries. Thus, pseudocapacitors can undergo a large number of charge/discharge cycles without significant degradation, thanks to the reversible nature of the redox reactions [31]. As electrode materials, a variety of structures can be used for the construction of pseudocapacitors, including transition metal oxides and conducting polymers, which offer high pseudocapacitance due to their ability to host faradaic reactions. The materials typically possess a high specific capacitance, quick charge/discharge rates, and excellent cycling stability, making them suitable for a variety of applications. The incorporation of ions into the structure of the materials, as well as the formation of species that link to the surface, contribute to energy storage. Thus, if the redox process is reversed, the electrode materials return to their initial state during discharge, and then the stored charge is discharged. Even if a significantly higher specific capacitance at a lower cost occurs when the oxide-based materials are compared to carbon materials, due to the specific properties, such as higher density and gravimetric capacitance, the oxide materials are profitable for the construction of the devices with high volumetric capacitance. Metal oxides, mixed metal oxides, and conducting polymers are the basic materials used in achieving pseudocapacitors. The three main types of electrochemical charge transfer are applied in pseudocapacitors, such as adsorption of ions on the electrode surface, oxidation-reduction reactions, and doping of conducting polymers.

### 2.3. Symmetric, Asymmetric, and Hybrid Supercapacitors

Supercapacitors are classified as symmetric, asymmetric, or hybrid based on electrode configuration. A symmetric supercapacitor possesses two similar electrodes, while an asymmetric one has two dissimilar electrode materials, and a hybrid supercapacitor has two dissimilar electrodes, one for a battery and one for a capacitor (Figure 3) [18]. Usually, in symmetrical capacitors, the electrode has the same mass charge and they are made of EDL-type or faradic-type materials. For example, Jahdaly et al. made a symmetric hybrid device AC/Co_3_O_4_//AC/Co_3_O_4_ based on abundant and natural marine red algae extract, resulting in a time- and energy-efficient process. This electrode displayed specific capacitances of 202 F g^−1^ at 0.5 A g^−1^. Further, this device achieves an energy density of 19.16 Wh kg^−1^ even at a high-power density of 1.91 kW kg^−1^ [32]. Zhao et al. used a facile synchronous activation and loading approach to synthesize HPC-MnO composite materials employing waste biomass litchi shell as a carbon source and KOH-KMnO_4_ as a chemical co-activation agent. This symmetric hybrid device exhibited an energy density of 57.7 Wh kg^−1^ at 400 W kg^−1^ power density. This outstanding performance was attained by combining the electrochemical double-layer capacitance of porous carbon with pseudocapacitance from manganese oxide redox processes [33].

Instead, the asymmetric supercapacitors use EDLC and pseudocapacitive electrodes composed of one or more of the same materials with varied mass loading.

Hybrid capacitors combine both types of materials (EDL and pseudocapacitive) and store charges using electrostatic and electrochemical methods. Due to its asymmetry, the hybrid supercapacitor is able to provide high energy and power density. The anode of an asymmetric hybrid supercapacitor is made of a pseudocapacitive material with a high specific capacitance that stores a large amount of charge in a reversible redox reaction due to the faradaic activities taking place there. Often the cathode is constructed from high surface carbon, such as activated carbon or graphene, to achieve a high electrochemical double-layer capacitance. EDLC keeps the charge at the negative electrode by adsorbing ions onto the electrode–electrolyte interface. and an electrolyte separates the anode and cathode, allowing ions to move freely between them while maintaining a uniform charge/discharge ratio. One example is presented by Yin et al., which uses metal oxides and carbon material CC/SnO_2_/MnO_2_/NiO/Ni foam to design and synthesize a novel alkaline asymmetric supercapacitor device. This device delivers a high energy density (64.4 Wh kg^−1^) at a high power density (250 W kg^−1^), good cycle stability, and high environmental suitability [34]. Lee et al. successfully developed an asymmetric supercapacitor using MoO_3_@CNT and MnO_2_@CNT composites as negative and positive electrodes. These devices exhibited high energy and power densities of 27.8 W h kg^−1^ and 524 W kg^−1^, and excellent cycle stability at an operation voltage window of 2 V in the Na_2_SO_4_ aqueous electrolyte [35].

Recent research has focused on developing hybrid electrochemical capacitors (HECs) that store charges asymmetrically and simultaneously using surface ion adsorption/desorption on the cathode and lithium/sodium intercalation on the anode. HECs have higher energy density than EDLCs and higher power density than conventional battery systems. An HEC using a carbon type for the negative electrode combined with activated carbon as a positive electrode is a promising power source for hybrid electric vehicles and fuel cell electric vehicles due to the higher energy density [36]. For example, Wang et al. created a Li-HEC, the porous carbon material was prepared from silkworm excrement by utilizing its highly porous and three-dimensional open structure. The resulting carbon material has a surface area of 2826 m^2^ g^−1^. They combined PC-SE with Si/C to make a Li-HEC, which demonstrated exceptional energy density (242.2 Wh kg^−1^ at 325.3 W kg^−1^) and stability during repeated charge and discharge cycles [37]. In terms of metal oxides, Zhang et al. have made a hybrid battery device based on Co_3_O_4_/N-doped activated carbon that has achieved high energy densities in a broad range of power densities, e.g., 76.7 Wh kg^−1^ at 0.29 kW kg^−1^, 46.9 Wh kg^−1^ at a high-power density of 18.7 kW kg^−1^, outperforming most of the literature reported hybrid supercapacitors [38].

## 3. Electrode Materials

The selection of electrode materials is essential for the supercapacitor system and should possess some unique properties such as high conductivity, better resistance to temperature changes, large specific surface area, and environmental compatibility. Moreover, these characteristics are strongly influenced by the interactions between of the supercapacitor components (anode, cathode, and electrolyte). A supercapacitor’s performance is determined by its electrode material’s ability to conduct faradaic charge transfer smoothly [39]. The energy storage devices manufactured on the basis of transition metal oxides (TMO) show excellent performance because these oxides present many very important characteristics: wide bandgap, improved reactivity, and chemical stability, very good electrical conductivity, etc [40]. To increase the efficiency of materials, there are many studies in which TMOs are combined with other transition metals, metal oxides, carbon-based materials, etc. Thus, the specific surface increases, and the intercalation/deintercalation of ions and the conductivity improve considerably [41]. Various metal oxides are used for the manufacture of the supercapacitor electrodes, such as TiO_2_, NiO, ZnO, Fe_2_O_3_, MnO_2_, RuO_2_, IrO_2_, MnO_2_, ZnO, etc [42]. In this study, our focus will be on the following metal oxides, TiO_2_ and Fe_2_O_3_ as TMO, Ga_2_O_3_, and ZnO as NTMO, due to good capacitance and also for super capacitive applications.

### 3.1. TiO_2_

Compared to other metal oxides such as MnO_2_, Co_3_O_2_, and RuO_2_, TiO_2_ used as electrode material for electrochemical energy storage devices (supercapacitors or batteries) recently gained more attention due to its good chemical stability, low cost, availability in abundant amounts, environmental friendliness, and multiple oxidation states [43]. Based on its special physicochemical properties, the large specific surface area of TiO_2_ has gained special attention due to its ability to provide the highest performance for a variety of applications. However, its electrical conductivity is poor, so this impediment limits the application of TiO_2_ for high-performance supercapacitors [44]. To overcome these defects, a new strategy has been carried out to increase the specific capacitance of TiO_2_ by coupling with other materials. Based on recent studies, in order to improve the electrochemical performance, TiO_2_ nanoparticles have been coupling with MoO_3_ through the construction of Ti-O-Mo bonds instead of metal-metal bonds [45]. S. Sun et al. have synthesized MoO_3_−x/TiO_2_ electrode composites by galvanostatic deposition and annealed at 550 °C in Ar atmosphere. This electrode has been prepared to obtain the highest specific capacitance of about 24.74 mF cm^−2^ at a current density of 0.4 mA cm^−2^ and good capacitance retention (86.6%), even after 1000 continuous charge/discharge cycles in Na_2_SO_4_ electrolyte [46]. In another study, B. Ezhilmaran and S.V. Bhat, a TiO_2_/α-MoO_3_ bi-layer electrode was designed, which demonstrated that this electrode exhibits the efficient intercalation/de-intercalation of Al^3+^ ions in an electrochromic supercapacitor. A stable performance with TiO_2_/α-MoO_3_ bi-layer electrode was obtained in terms of coloration efficiency (128 cm^2^ C^−1^), transmittance change (54%), switching time (~1 s), and a real capacitance (218.8 mF cm^−2^) [46].

Another promising material for supercapacitor electrodes was the combination of TiO_2_ with V_2_O_5_ due to the improvement of chemical stability and electrochemical activity by reducing the resistance to intra-particle electron hopping. Therefore, TiO_2_-V_2_O_5_ nanocomposite is one of the most exceptional candidates because of its high capacitance, low cost, non-toxicity, and easy accessibility to nanostructures [47]. Xu J. et al. have reported a heterojunction composite of V_2_O_5_ nanobelt arrays/TiO_2_ nanoflake arrays grown directly on nickel foam. They showed that TiO_2_/V_2_O_5_ composite has a high specific capacitance of 587 F g^−1^ at a current density of 0.5 A g^−1^, a high coulombic efficiency (>92%), excellent cycle stability (92%) after 5000 cycles, and a low charge transfer resistance of 2.6 Ω. The high supercapacitive performance was attributed to their direct growth on the conductive current collector and the synergistic effect of V_2_O_5_ and TiO_2_ [48]. Zhang L. et al. prepared a hierarchical structure of the TiO_2_ nanorod/MnO_2_ ultrathin nanosheet core/shell nanocomposite on FTO substrate (with current collector function) using two easy steps by hydrothermal treatment followed by annealing of the obtained electrode and thus avoided the morphology damage, pore blocking, and reduced conductivity of electrode materials, which is favorable for electrochemical behavior [49].

### 3.2. Fe_2_O_3_

As well as TiO_2_, Fe_2_O_3,_ especially in hematite form, α-Fe_2_O_3_, is often used in supercapacitor applications due to its specific capacitance, low cost, abundance in nature, environmental friendliness, thermal stability, and corrosion resistance [50]. Among all the crystalline forms of Fe_2_O_3_, α-Fe_2_O_3_ is one of the most stable corundum-like hexagonal structures [51] and has proved to be a potential candidate for multifunctional applications, including rechargeable batteries [50]. Fe_2_O_3_ can be used as a potential negative electrode in obtaining capacitors [52]. Based on the electrolyte solution, cell design, structure, and morphology, the two forms of Fe_3_O_4_ or α-Fe_2_O_3_ show good pseudocapacitor behavior in alkaline electrolytes with capacities between 5–500 F g^−1^. However, due to poor electrical conductivity and metastability, the high-rate charge/discharge performance is not satisfied [53]. Thus, to improve the properties of Fe_2_O_3_-based electrodes, composite materials were obtained, and an improvement in electrochemical performance was observed due to the increase in the number of active sites, implicitly a large active surface that is favorable for charge/discharge reactions [50]. Guo M. et al. have successfully synthesized Fe_2_O_3_@NiS/Co_3_S_4_ electrodes through an in-situ solution vulcanization process. Based on this designed electrode and active carbon as a negative electrode, the ASC device possesses a high specific energy density of 43.8 W h kg^−1^ at 810 W kg^−1^ and long-term cycling stability (92.4% capacitance retention after 10,000 cycles at 5A g^−1^) [54]. Guo G. et al. have prepared an electrode based on Fe_2_O_3_ material grown on Ni foam through a simple hydrothermal method. After performing some electrochemical tests, they found that the Fe_2_O_3_/MgFe_2_O_4_ sheet electrode has better cycling stability and multiplicative performance compared to the simple Fe_2_O_3_ sheet electrode. For a molar ratio 1:1 of Fe_2_O_3_ to MgFe_2_O_4_ material, the initial specific capacitance of the sheet electrode was 815 F g^−1^, and the capacitance remained at 81.25% of the initial specific capacitance after 1000 cycles [55]. Jiang H. et al. have synthesized through a facile hydrothermal method α-Fe_2_O_3_ nanosheet@Ni(OH)_2_ for electrode material of supercapacitor. This hybrid material exhibited a good rate capability with a specific capacitance of 356 F g^−1^ at a current density of 16 A g^−1^ and excellent cycling stability (a capacity retention of 93.3% after 500 circles) [56]. In another study, Nicolaescu M. et al. developed a flexible negative electrode for supercapacitors, based on amorphous iron-based ribbons decorated with nanostructured iron oxides. The CV curves show that both samples functioned as negative electrodes and a charge storage capacity of 16.25 F g^−1^ was measured for the sample treated in 0.2 M NaOH solution and 19.5 F g^−1^ for the 0.4 M sample at a scan rate of 0.05 V s^−1^. Thus, according to the obtained experimental data, amorphous ribbons decorated with Fe_2_O_3_ nanoparticles have a high potential in supercapacitor applications [57].

### 3.3. Ga_2_O_3_

Recently, Ga_2_O_3_ has attracted scientific attention due to its applications in electronics, optoelectronics, memory, sensing systems, deep ultraviolet transparent conductive oxide electrodes, photocatalysts, and so on. Ga_2_O_3_ exists in six different polymorphs such as α, β, γ, δ, ε, and κ, of which the β-phase is the most stable, while the κ-phase is transitory [58]. To improve the electrochemical characteristics of Ga_2_O_3_, it can be combined with other oxides or metals as follows. Fe_2_O_3_/Ga_2_O_3_ composite and GaFeO_3_ electrodes worked as rechargeable electrode materials for lithium-ion batteries, whereas their capacities gradually decreased with increasing cycle numbers. The initial Li insertion capacities (cut-off voltage: 0.01 V) were 1643 mA hg^−1^ for Fe_2_O_3_/Ga_2_O_3_ composite and 1196 m hg^−1^ for GaFeO_3_, respectively. Despite the same Fe/Ga atomic ratio, the Fe_2_O_3_/Ga_2_O_3_ composite showed a higher capacity than that of GaFeO_3_ over the 50 cycles [59]. Xu H. et al. prepared two-dimensional SnO_2_-Ga_2_O_3_ n-p heterostructures and an improvement in electrochemical performances was observed. Thus, the value of the specific capacity was ~167 F g^−1^ at the current density of 7.69 A g^−1^, as well as excellent capacity retention (~92.55%) after 10,000 continuous cycles [60]. Hu Y.-L. et al. synthesized a hybrid structure of GaN/Ga_2_O_3_ on carbon cloth used in assembling a symmetrical supercapacitor. Thus, both the electron density and specific capacitance of the composite electrode significantly improved, providing an energy density of 0.58 W h kg^−1^ (3.54 mW h cm^−3^) with a power density of 154 W kg^−1^ (0.94 Wcm^−3^) [61]. Another study showed that the aqueous symmetrical Ga_2_O_3_/CC supercapacitors exhibited good electrochemical performance, with a specific capacitance of 1394 mF cm^−2^ at 0.5 mA cm^−2^, retaining 67% at 20 mA cm^−2^, and an energy density of 26.26 μW h cm^−2^ at a corresponding power density of 0.097 mW cm^−2^ and 76.5% capacity retention after 20,000 cycles at 10 mA cm^−2^. The obtained results are encouraging and offer an opportunity for the development of supercapacitors based on α-Ga _2_O_3_ in the future [62]. Yang S. et al. have developed the anodes for Li-ion batteries based on hybrid materials of Li_3_VO_4_ and Ga_2_O_3_ nanoparticles embedded in nitrogen-doped carbon. Thus, the results obtained showed that electrodes presented a current of 0.1 A g^−1^, providing a reversible capacity of 652.6 mA h g^−1^ after 400 cycles and a discharge capacity of 750.3 mA h g^−1^ [63].

### 3.4. SnO_2_

Due to its low cost, eco-friendliness, non-toxicity, and good chemical stability, SnO_2_ nanostructures are suitable for potential application in many fields of energy storage and conversion [64]. SnO_2_ is an important n-type wide-bandgap semiconductor that possesses a high electrical conductivity (21.1 Ω.cm), high theoretical capacity (~782 mA h g^−1^), and superior electron mobility (100–200 cm^2^ V^−1^ s), making it a promising choice for supercapacitor application [65]. Shin et al. produced a hierarchical SnO_2_ core-shall nanobranches through thermal oxidation, achieving a specific capacitance of 40.5 μFcm^−2^ and long-term cycling stability up to 1000 cycles with the loss of only 8.9% of the maximum specific capacitance [66]. Akkinepally B. et al. investigated SnO_2_-decorated ZnO hexagonal prisms as supercapacitor electrodes synthesized for building cost-effective energy-storage devices. The SSC device exhibited a specific capacitance of 83 F g^−1^ at 1.2 A g^−1^; impressive power and energy densities were achieved by the device with values between 2808 and 70.2 W kg^−1^. This SSC device maintained a capacity preservation of 75% throughout 5000 galvanostatic charge/discharge sequences [67].

To improve the performance of SnO_2_ as a supercapacitor, many efforts have been carried out by doping SnO_2_ with other elements [68]. For example, Yin and Guo prepared Pd-loaded and Fe-doped SnO_2_ by a simple sol–gel method, when the amounts used were 10 mol% Fe and 0.2 mol% Pd, the composite presented the highest sensitivity and selectivity to CO in the range of 200–3000 ppm at 350 °C [69]. The response value of the composite material to 2000 ppm CO was raised 13 times compared with pure SnO_2_. Turgut et al. deposited Mo/F double-doped SnO_2_ films by spray pyrolysis technique [70]. The electrical and optical studies suggest that the Mo/F double-doped films are very attractive candidates for several technological applications. Also, Wang et al. [71] have reported the synthesis of SnO_2_ nanoflowers using NaF as the morphology-controlling agent and SnCl_2_·2H_2_O as the tin source. Based on the obtained results, the authors highlighted the possibility of combining both the surface and electronic properties of the composite materials based on doped SnO_2_ for improved sensing characteristics.

## 4. 2D and 3D Supercapacitors Materials

The advancement of 2D and 3D nanostructures has significantly transformed supercapacitors, bringing significant improvements in energy storage capabilities. Supercapacitors are known for their high-power density, quick charge/discharge cycles, and long lifespan, and are strongly influenced by the properties of the nanomaterials. TMO and PTMO materials are particularly interesting for supercapacitor applications because of their high surface-to-volume ratio, theoretical specific capacitance, electrochemical stability, natural abundance, simplicity, and chemical stability [72]. Incorporating the 2D and 3D structures of metal oxides and other classes of materials enhances the surface area, conductivity, and overall electrochemical performance, such as high theoretical specific capacitance of supercapacitor electrodes, significantly improving their efficiency and effectiveness [73,74]. 2D nanostructures, including nanosheets, nanoflakes, nanoplatelets, thin films, and materials such as graphene, transition metal dichalcogenides, MXenes, layered double hydroxides, graphitic carbon nitride, metal-organic frameworks, black phosphorus, enhance the performance of SCs. In recent years, metal oxide layers and 2D structures of oxide composites have been shown to improve the performance of SCs by enhancing numerous electrochemically active sites, high planar electrical conductivity, and large surface areas [75]. These oxide nanomaterials can be integrated into hierarchical structures with other 1 and 2D materials. The atomic-scale thinness of these materials shortens the ion diffusion channels and reduces the ion diffusion resistance, which improves the efficiency of charge storage and transfer. The distance between nanosheet layers resists bending and folding, making them ideal for flexible SC applications. However, some of the main engineering problems are due to the high aspect ratio and tendency to restack and aggregate. These behaviors reduce the accessible surface area and have a negative impact on electrochemical performance, due to intense Van der Waals forces and interlayer contact, leading to further shrinkage and degradation of SC performance.

**Figure 4:**
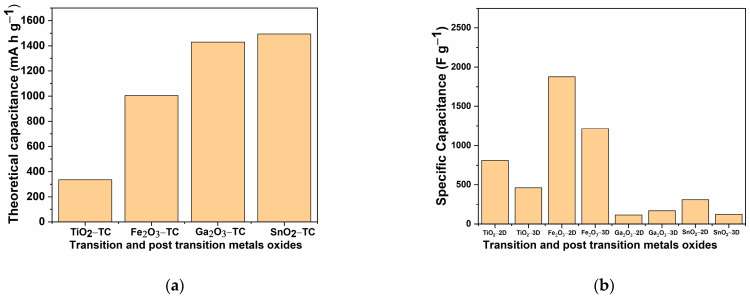
(**a**) Theoretical specific capacitance of some transition and post transition metal oxide for TiO_2_ [77]; Fe_2_O_3_ [78]; Ga_2_O_3_ [79] and SnO_2_ [80]; (**b**) Specific capacitance of 2D and 3D structures of some transition and post transition metal oxides: 2D-TiO_2_ [81]; 3D-TiO_2_ [77]; 2D-Fe_2_O_3_ [82]; 3D-Fe_2_O_3_ [54]; 2D-Ga_2_O_3_ [83]; 3D-Ga_2_O_3_ [60]; 2D-SnO_2_ [84]; 3D-SnO_2_ [85].

On the other hand, 3D nanostructures including 3D carbon materials, 3D vertically aligned nanomaterial (nanotube, nanowire, nanosheet), 3D porous current collector (foam and sponges’ templates), 3D foam, hydrogels, aerogels, sponge nanostructures, polyoxometalate-based 3D compounds, 3D microstructure from 3D printing process, 3D fabric-based structures, 3D porous heterostructures (oxide/carbon composite) can greatly improve the energy storage, both as 1D and 2D nanomaterials [76,77]. Notably, 3D nanostructures with porous and hollow designs, such as hollow metal oxides, are highly effective because they facilitate the transport of electrolyte ions, thus increasing the surface area available for redox reactions. Metal oxide materials incorporate multiple metal cations and exceed an electrochemical capacity of the pristine metal oxide because of the increased number of electroactive sites that generate an enhancement of ion transport generated by the additional metal ions. Also, the doping with metal ions shows that the introduction of multivalent and multicomponent metal oxides enhances the electrochemical performance of SCs. A theoretical specific capacitance for the selected metal oxide TiO_2_, Fe_2_O_3_, Ga_2_O_3_, and SnO_2_, and the specific capacitance for 2D and 3D structures of transition and post transition metals oxides, are graphically presented in Figure 4a,b.

### 4.1. Synthesis Rout for Obtaining 2D and 3D Materials

**Figure 5:**
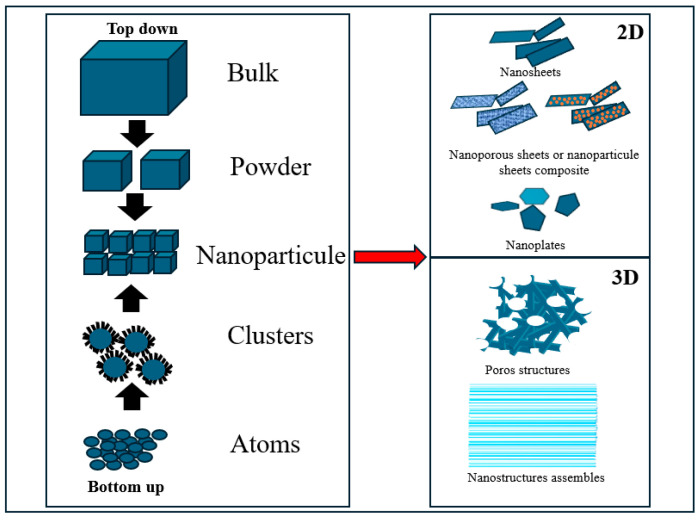
The schematic representation of the top-down and bottom-up approaches in synthesis of 2D and 3D materials.

The intensive development of nanomaterials in recent periods due to intense demand has opened new perspectives in many fields such as electronics, optoelectronics, chemical catalysis, medicine, and many others. Their unique properties and enhanced characteristics generated by the small size provide a significant improvement for the future innovation of these materials. A specific area of interest over the years has been the development of nanomaterials for supercapacitors due to their high surface area and exceptional conductivity that can significantly improve their performance [86]. There are two main approaches in the synthesis of nanoparticles for supercapacitor applications: the top-down and the bottom-up approach [87]. In Figure 5, both approaches used to synthesize 2D and 3D structures for SC applications are graphically presented.

In the top-down approach, bulk materials are reduced in size to obtain nanoparticles in mono- and multi-layer forms, forming 2D and 3D structures. This requires the use of external influences, using various techniques, such as lithography, milling, laser ablation, sputtering, and dealloying. These are effective techniques for creating the fine structure required for high-performance supercapacitor materials [87].

**Figure 6:**
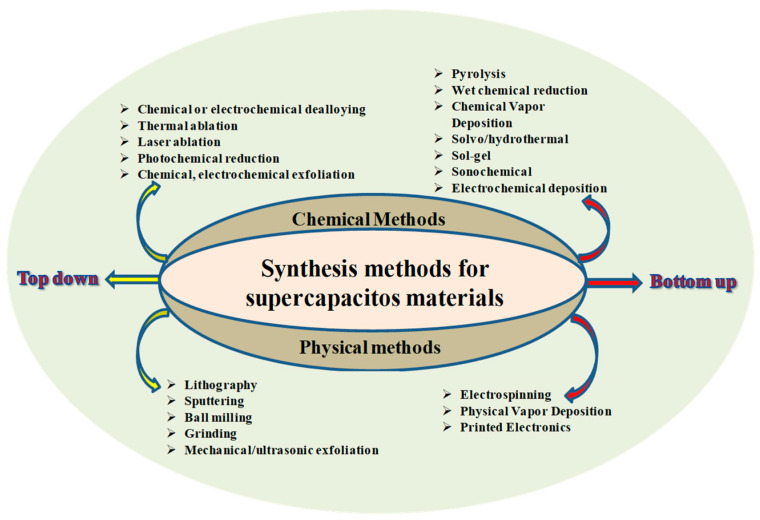
The main synthesis process for fabrication of supercapacitors materials.

In the synthesis of nanomaterials, the bottom-up approach involves the assembly of nanoparticles using processes such as atomic or molecular components through physical, chemical, or biological methods, chemical and physical synthesis being the most common. This approach enables precise control over nanoparticle properties such as size, shape, and surface chemistry interaction, which are essential for optimizing SC performance. Although the potential costs and environmental concerns associated with these methods are relatively high, they are very effective for producing large quantities of nanoparticles with well-controlled properties. In addition, atoms or molecules can be grown or assembled along a specific direction on a suitable substrate in a controlled environment, eventually forming an ultra-thin 2D structure or a more complex 3D structure [86]. Figure 6 provides a schematic representation of the top-down and the bottom-up approach regarding different methods of obtaining nanostructured oxides through chemical and physical synthesis approaches.

#### 4.1.1. Top-Down Approach

Lithography is one of the most used techniques in microfabrication for modeling nanomaterials with high precision. It involves transferring a pattern from a mask to the surface of a substrate mainly using light. This is essential for creating high resolution 2D structures and can be adapted to form 3D nanostructures when combined with other techniques. In supercapacitors, lithography is used for miniaturized applications mainly in the fabrication of MSCs. This technique enables the precise fabrication of electrode materials with well-defined nanoscale shapes and sizes [88]. Pitkänen et al. created an MSC for on-chip energy storage using photolithography. They used Pt and Mo layers as current collectors and a 3D highly aligned vertical carbon nanotube (CNT) electrode decorated with MnOx nanoparticles (as active material), achieving a maximum areal capacitance of 37 mF cm^−2^ with an in-plane electrode design. The on-chip integration is scalable, compatible with standard CMOS processes, and provides lightweight energy storage essential for portable and autonomous devices [89].

Ball milling is a mechanical process that involves grinding the bulk materials into finer particles using a spherical grinding ball in a rotating cylindrical chamber. This technique is very effective for breaking down bulk materials into nanostructures by mechanical force. Ball milling is a well-used industrial technique that can produce nanoparticles with controlled sizes and shapes, which is very useful in carbon-based EDLCs. The particle size distribution of activated carbon is one of the most important parameters for increasing the performance of highly porous ACs in EDLC [90]. Additionally, ball milling is widely researched for the fabrication of 3D porous structures and 3D composites for supercapacitors, and it is a common method for post-processing materials for 3D printing [91]. The method is very scalable and cost-effective, making it suitable for the large-scale production of nanomaterials. Although the ball-milling method is widely used to create advanced carbon composites, S. Liu et al. used this process to fabricate PPy/Ni_2_P/GO composites for high-performance supercapacitor electrodes. These electrodes exhibited a high specific capacitance of 741.5 F g^−1^ at 1 A g^−1^, good rate capability of 81.4% capacitance retention at 10 A g^−1^, and long-term cycling stability 89.76% retention after 5000 cycles at 1 A g^−1^. Applied in an asymmetric supercapacitor, the PPy/Ni_2_P/GO//AC device demonstrated an energy density of 61 W h kg^−1^ at a power density of 849.8 W kg^−1^ and excellent cycling performance, with 89.4% capacitance retention of 89.4% after 3000 cycles at 10 A g^−1^ [92].

Mechanical exfoliation is a relatively simple process that aims to peel a few graphene layers from bulk 3D graphite material by overcoming the Van der Waals attraction force between the graphene flakes. This is a typical mechanical technique using either normal force or lateral force. Normal force is applied using Scotch tape micromechanical cleavage, which can effectively separate graphite layers [93]. These advances contributed to winning the Nobel Prize in Physics in 2010. The mechanical mechanisms involved in exfoliation are shear or normal force generating mechanisms. These methods are involved in the reported exfoliation techniques for graphene production. By perfecting these mechanical approaches and controlled exfoliation of graphite, obtaining high-quality graphene with high efficiency becomes feasible. Yi M. and Shen Z. presented a comprehensive review of mechanical exfoliation for scalable graphene production, highlighting various methods used in the production of graphene by mechanical exfoliation [94]. In another study, Thaneswari S.A. et al. developed a single-step method for manufacturing a few-layer graphene using a kitchen blender. The synthesis material was functionalized for supercapacitor applications. The maximum capacitance was calculated to be 33 F g^−1^ at a current density of 1 A g^−1^ [95]. After the successful exfoliation of graphene from graphite, this method is also employed to synthesize 2D nanosheets from other materials for supercapacitor applications [96,97]. This approach is simple, scalable, and maintains high crystallinity, but does not allow precise control over the thickness and size of the nanosheets.

Ultrasonic exfoliation is a widely used method for the mass production of 2D materials. This liquid-phase technique treats layered crystals with ultrasound in stabilizing liquids. Ultrasonic waves break the Van der Waals forces between layers without affecting the covalent bonds within the layers. The mechanical energy generated by the ultrasound creates cavitation bubbles around the surface of bulk material. The resulting waves generated a separation of layers, producing a dispersion containing 2D nanosheets. The choice of solvent is crucial for the stability and ratability of the exfoliated nanosheets [98]. Surfactants or polymers provide stable repulsive forces that match the surface energy or solubility parameters of the nanosheets, preventing aggregation [99]. The work of Zainab et al. was focused on optimizing the time of the exfoliation process of graphite to fabricate graphene/GO/GrO supercapacitors. They demonstrated that a sonication period of 48 h resulted in a notable specific capacitance of 534.53 F g^−1^ at 1 A g^−1^ and an energy density of 66 kW kg^−1^. The power density increased from 0.75 kW h kg^−1^ to 7.5 kW h kg^−1^ as the current density decreased from 10 to 1 A g^−1^ [100]. K. Zhang and coworkers used a mechanical exfoliation technique based on a high-pressure shear exfoliation method to obtain a few-layer graphene which improved the specific capacitance at 135 F/g compared to the above study [101].

Chemical exfoliation transforms layered bulk crystals into monolayers or a few layers using intercalators such as acids, bases, inorganic salts, oxidizing agents, and reactive molecules. This method disrupts chemical bonds in the basal plane of 2D nanomaterials by introducing heteroatoms or functional groups, facilitating cleavage, preventing reaggregation, and improving functionality [102]. Tomy M. et al. presented the impact of chemical exfoliation on the specific capacitance of composite electrodes for supercapacitor applications. Their research shows that the chemically induced exfoliation improved the energy storage performance of MoS_2_@PANI electrodes. The heterostructure composite of MoS_2_ex@PANI generated a direct correlation between exfoliated MoS_2_ and PANI. Finding the right parameters generated a considerable improvement in capacitance. The combined pseudo-capacitance and electric double-layer capacitance mechanisms resulted in an enhanced specific capacitance of 277 F g^−1^ in a 1 M H_3_PO_4_ electrolyte, surpassing the performance of the non-exfoliated composite electrode [103].

Electrochemical exfoliation is the most widely used method, differing from the above method by using electric currents to transfer molecules or ions into the interlayer space, thus increasing the distance between the layers. This is environmentally friendly as the electrolyte is recyclable and a reduction of synthesis time is usually achieved, unlike in other methods. However, its major disadvantages are the poor scalability in the industrial sectors [86]. Zheng and his team used electrochemical exfoliation of black phosphorus followed by in situ growth of nickel–cobalt hydroxide to form a 3D NiCo-LDHs@BP heterostructure. The resulting 3D electrode exhibited a substantial specific capacitance of 1291.3 F g^−1^. In addition, the solid-state flexible asymmetric supercapacitors demonstrated a high specific energy density of 279.6 µW h cm^−2^. The stability is presented by maintained a decrease of capacity of 85.6% after 5000 folding cycles. This research innovation has successfully powered wearable devices such as watches and heart rate monitors [104].

Chemical or Electrochemical Dealloying involves selectively removing one or more components from an already fabricated metal alloy, resulting in a porous and nanoporous structure [105]. In chemical dealloying, the as-produced alloy is immersed in a corrosive chemical solution that dissolves (dealloying) the less noble metal or metals, leaving behind a nanoporous framework. Electrochemical dealloying works differently by using an electrochemical cell in which an enhanced electrical current is typically used to selectively dissolve certain elements in the alloy [106]. This technique is particularly efficient for creating 3D porous structures with large surface areas, creating ion accessibility and storage capacity in supercapacitors. Deposition of active materials on the surface of the nanoporous metals further improves their performance, like supercapacitor materials. This improvement is associated primarily with the generation of many active sites between substrate and active material. Dealloying offers simplicity and scalability and is shown to generate better control over the microstructure. The as-synthesis nanoporous materials from this process have unique 3D bicontinuous topologies [107]. The dealloying technique, traditionally known for the synthesis of 3D materials, has recently expanded to produce 0D, 1D, and 2D materials for energy storage applications [108]. Qiu et al. prepared hierarchical nanoporous nickel alloy electrodes using a Mn-Cu-Ni alloy as the precursor. Manganese was removed in a weak acid solution, and the resulting material was annealed at high temperatures under an inert gas. They reported a high specific capacitance of 1900 F g^−1^, approaching the theoretical value for Ni(OH)_2_. This high performance is likely attributed to the nanoporous structure of the alloy [109].

**Figure 7:**
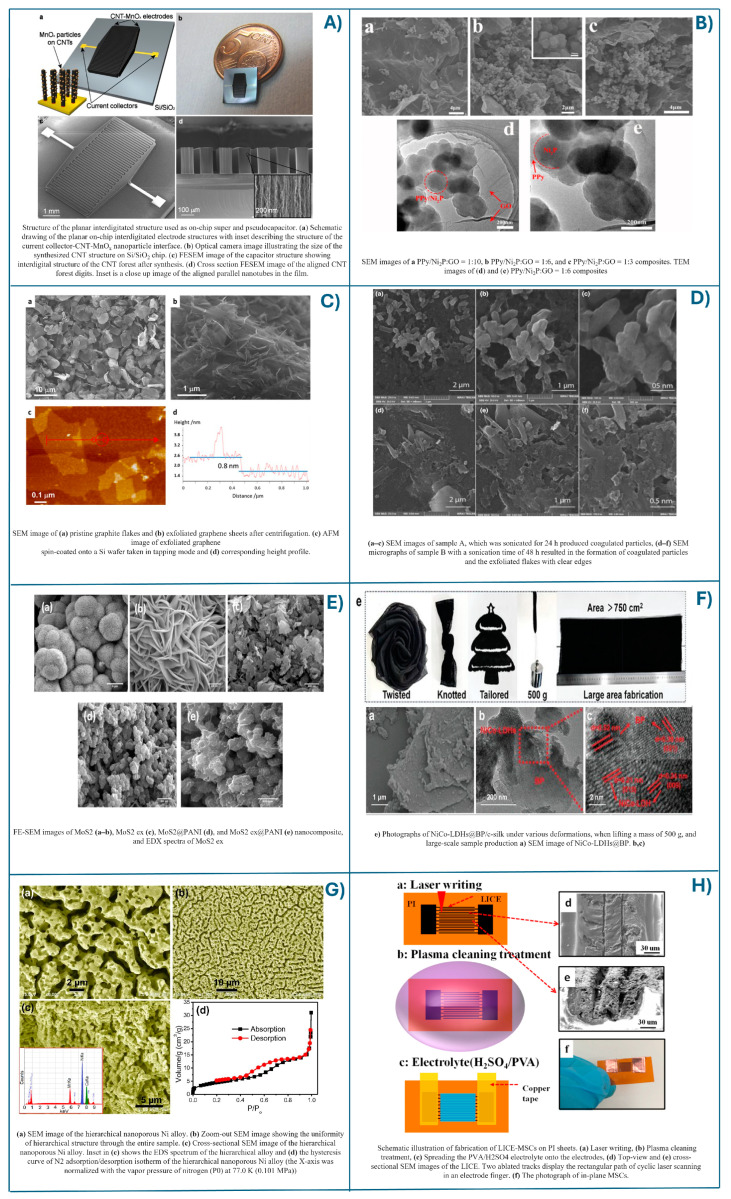
Different surface morphology and electrode fabrication for 2D and 3D supercapacitors from top-down approach: (**A**) Reprinted with permission from [89]. Copyright 2017, Springer Nature; (**B**) Reprinted with permission from [92]. Copyright 2021, Springer Nature; (**C**) [101]; (**D**) [100]; (**E**) Reprinted with permission from [103]. Copyright 2023, Springer Nature; (**F**) Reprinted with permission from [104]. Copyright 2024, Wiley; (**G**) Reprinted with permission from [109]. Copyright 2014, Elsevier; (**H**) Reprinted with permission from [113]. Copyright 2017, Elsevier.

Laser ablation is a well-known process in which a laser beam is focused on the surface of the material to create nanostructures and patterns with precisely controlled sizes. Laser ablation can be performed in different environments, such as vacuum, gas, or liquid, which can influence the resulting nanostructures. It is also one of the most popular techniques for the synthesis of carbon-derived nanomaterials. Laser ablation synthesis in solution is a very promising method for creating nanoparticles in a liquid medium. This technique uses established laser-based technologies to produce additive-free nanocolloids that are less harmful and more environmentally friendly [110]. Taccogna F. presented the mechanism that takes place during laser ablation very well. When a solid target is irradiated with a laser pulse, the absorbed energy expels material as a plasma plume, which is confined by the surrounding liquid’s pressure, generating a cavitation bubble. This bubble collapses, releasing charged nanoparticles by collecting electrons that attract ions to maintain equilibrium; their size is influenced by plasma parameters such as density and temperature. The liquid’s confining effect on the plasma plume leads to small nanoparticles with a negative equilibrium charge [111]. In supercapacitors, the laser ablation approach is used to synthesize carbon-derived nanomaterials. This involves the laser carbonization of polyimide material in air atmosphere, resulting in carbon-derived nanomaterials for use in supercapacitors. This approach is particularly interesting for MSCs because by controlling the laser direction, different high-precision laser-induced graphene patterns can be achieved on the polyimide surface, allowing the current collector and active material to be obtained in a single process without affecting the unexposed areas or requiring lithographic masks. The most commonly used polyimide is Kapton material, and many researches have been focused on studying its structure as a function of the level of ablation (laser power) to enhance the electrical conductivity of the samples [112]. An in-plane and multi-layer 3D-MSCs based on laser carbonization of polyimide (PI) sheets was successfully manufactured by Shutong W. et al. Femtosecond laser pulses convert insulating polyimide into a conductive porous carbon structure, achieving a specific capacitance of 22.40 mF cm^−2^ for a single-layer supercapacitor at 0.1 mA cm^−2^. Stacking layers increases capacitance to 37.2 mF cm^−2^ and 42.6 mF cm^−2^ for 2-layer and 3-layer devices, respectively. They also demonstrated that scaling up voltage and capacitance is feasible by arranging the supercapacitors in series or parallel arrays [113]. Different morphologies can be obtained by applying the top dawn method for electrode fabrication with 2D and 3D structures for supercapacitors, as shown in Figure 7.

#### 4.1.2. Bottom-Up Approach

Electrospinning is a widely used method to produce a micro and nano fibrous structure which can be incorporated in 2D and 3D supercapacitors. Nanofiber fabrication by electrospinning involves stretching a viscoelastic solution using electrostatic forces. This technique uses a high voltage applied to both the solution transmitter and the receiver, generating a jet of liquid. The electrospinning setup includes a high-voltage source, a syringe containing the spinning solution, and a collector for forming nanofiber films. The charged droplets appear at the needle tip due to the voltage, and when the electrostatic force surpasses the liquid’s surface tension, a Taylor cone forms, and the electric field stretches this cone into a fine jet. So, the rapid solidification result from the solvent evaporates and is collected as nanofibers structures [114]. Optimizing the morphology of electro-spun fibers is crucial for improving supercapacitor performance. This process has some key process parameters such as polymer concentration, applied voltage, flow rate, needle diameter, and needle-collector gap significantly influence the fiber characteristics [115]. For use in supercapacitors, 2D and 3D fiber materials are synthesized using sacrificial polymers as soft templates. Using this method, highly porous carbon nanofibers or fibrous oxides such as MgO, Fe_2_O_3_, SiO_2_, MnO_2_, and ZnO can be produced [116]. These hard templates are removed by etching using corrosive agents like NaOH, HF, or HCl [117]. Binitha G. and coworkers developed a straightforward synthesis method for two α-Fe_2_O_3_ nanostructures with different morphologies using electrospinning, integrating them into a 2D thin layer for supercapacitor applications, which achieved a high specific capacitance of 102 F g^−1^ at a scan rate of 1 mV s^−1^ [118]. In contrast, Najafi M. and his team demonstrated that α-Fe_2_O_3_/carbon composites (C-Fe_2_O_3_) offer superior supercapacitor performance due to their 3D electrode structure and higher mass loadings (>1 mg cm^−2^) on practical flat current collectors, eliminating the need for bulky porous substrates. The C-Fe_2_O_3_ electrodes exhibited a capacity of ~140 mA h g^−1^ (Ceff = 265.9 F g^−1^) at a scan rate of 2 mV s^−1^ [119].

Physical vapor deposition (PVD) involves vaporizing a base material and depositing a substrate in a vacuum chamber. Techniques such as electric arc evaporation, magnetron sputtering, and electron diffraction epitaxy are used to create 2D and 3D structures, making PVD particularly effective for thin-film fabrication. This process incorporated various vacuum deposition methods. This process is characterized by passing the material from a condensed phase to a vapor phase followed by a recondensation in a 2D thin film nanomaterial. The process involves spraying or evaporating the components to create a vapor phase, supersaturating this vapor in an inert atmosphere to facilitate condensation of the metal nanoparticles, and consolidating the nanocomposite by heat treatment in an inert atmosphere [120]. PVD is estimated to play a crucial role in manufacturing thin films for supercapacitors, which are essential for flexible and portable electronics. One of the most useful applications for the supercapacitor of this process is wearable energy storage devices due to the physical characteristics of 2D material synthesis in this process. In recent years, intense efforts have been made to develop flexible, stretchable, and portable rechargeable SCs. To assess their viability and portability, the researcher also focuses on mechanical tests combined with electrochemicals to ensure stability. Thin-film supercapacitors have emerged as promising energy storage devices due to their high power density, stability, lightweight nature, and ease of handling. However, traditional designs often suffer performance degradation due to exposure to the atmosphere and mechanical distortions, particularly in flexible systems. Flexible all-solid-state supercapacitors, considered state-of-the-art power supplies for miniaturized devices, effectively prevent harmful electrolyte leakage common in traditional aqueous electrolyte-based supercapacitors [121]. Achour A. and his team synthesized titanium nitride 2D thin films for micro-supercapacitors using the PVD process, studying the structural and morphological effect on capacitance. They demonstrated that titanium nitride films can have controlled porosity and can be relatively easily deposited on flat silicon substrates via reactive DC-sputtering. These films serve as high-performance micro-supercapacitor electrodes, achieving a superior volumetric capacitance of 146.4 F cm^−3^ and exhibiting outstanding cycling stability over 20,000 cycles [122].

Printed electronic (PE) techniques are an emerging class of technologies, such as inkjet printing, screen printing, and 3D printing, enabling the manufacturing of electronic devices at significantly reduced fabrication and prototyping costs. These techniques also allow the fabrication of devices on a large scale and use more unconventional substrates that have interesting mechanical properties. The 3D printing technology is an additive manufacturing category and is an advanced bottom-up manufacturing process. Using this process, 3D supercapacitor materials with complex geometry and different architectures can be manufactured. This technology offers significant advantages, including low operational costs and minimal material waste, making it a technology that has been used cost-effectively in recent years [123]. A 3D structure can enhance the electrode/electrolyte contact surface and improve charge storage processes and active mass utilization, increasing effective surface area. Additionally, a 3D porous current collector reduces resistance, leading to better electrochemical performance. As a result, the 3D supercapacitor shows a capacitance about 60 times higher than that of the 2D counterpart at a similar discharge rate [124].

Recently, there has been a growing interest in using MXene materials for 3D printed supercapacitors, leading to efforts in developing printable MXene inks for direct ink writing methods. Most of these efforts are focused on creating high-loading, additive-free aqueous MXene inks, such as Ti_3_C2Tx, for supercapacitor fabrication. Orangi J. and coworkers developed all-solid-state MSCs using 3D printing with additive-free, water-based MXene ink. MSCs leverage the high conductivity and electrochemical properties of Ti_3_C2Tx MXene and a 3D interdigitated electrode design to achieve high areal and volumetric energy densities. The process supports flexible MSCs on polymer and paper substrates, with printed devices showing impressive electrochemical performance, including a real capacitance of up to ~1035 mF cm^−2^, demonstrating Ti_3_C_2_Tx MXene as a superior electrode material [125].

The sol–gel method transforms a precursor solution into a gel following solidification by drying and heat treatment. This process allows precise control over the chemical composition and morphology. Following this process, a wide range of micro- and nano-structures can be synthesized like 2D and 3D materials. The 2D material included nanosheets, nanoplates, and nanopellets, but also self-assembled 3D morphologies such as flowers, hedgehogs, dandelions, and aerogels can be synthesized. As a low-cost, flexible, and well-established synthetic route, the sol–gel method enables the customization of the morphology, shape, size, homogeneity, and aggregation of the products by adjusting the synthesis parameters [126]. An interesting class of materials is considered aerogels, characterized by their high surface-to-volume ratio from nanometer-sized pores, low density, and high porosity for 3D supercapacitor applications. The term “aerogel” primarily describes the internal structure of the material, which allows the synthesis of aerogels from a wide variety of raw materials with different chemical properties [127]. Zhang Z. et al. synthesized monolithic NiO aerogels by the sol–gel method for use as supercapacitor electrodes. They show that the porous structure of the aerogel, supported with a nanoparticle, serves to enhance the active sites for pseudocapacitive reactions and ion transfer. They demonstrated that NiO aerogel electrode, annealed at 250 °C, achieved the best specific capacitance of 901 F g^− 1^ at 0.5 A g^− 1^ and a high-rate capability of 436 F g ^−1^ at 20 A g^−1^. The capacitance retention of 93% is achieved after 3000 cycles at 10 A g^−1^ [128]. Furthermore, in a more recent study, Ramkumar R. and his team synthesized NiO/Ni aerogels with a specific capacitance of 1060 F g^−1^ at 1 g^−1^ current density and 92% capacitance retention after 10,000 cycles [129].

The solvo/hydrothermal method uses solvents at controlled temperature and pressure to determine crystal growth and to form advanced nanomaterials. Technically, the hydrothermal method is more widely used because it involves only water as a solvent for synthesizing materials through a continuous reaction. Both processes exhibit similar mechanisms, require the same equipment, and facilitate the creation of complex 2D and 3D structures. By adjusting the synthesis parameters, micro/nanomaterials with various morphologies can be synthesized. The main advantages of this process for the synthesis of supercapacitor electrode materials are that they have micro/nano dimensions and offer short transmission distances of the active material, as well as large specific surfaces [130]. Both methods facilitate the one-step synthesis of 2D and 3D materials for supercapacitor applications with high capacitance. Li Y. and team used the hydrothermal method to fabricate a novel CuS and carbon dot decorated Ti_3_C2Tx composite in one step. The optimal C-Ti_3_C2Tx/CuS composite electrode achieved an excellent specific capacitance of 1186 F g^−1^ at a current density of 1 A g^−1^, significantly higher than Ti_3_C2Tx samples decorated with only CuS or CDs. This demonstrates the outstanding synergistic enhancement effect of CuS and CDs on the electrochemical performance of MXene. These results show that the hydrothermal process can produce high-performance 2D-Ti_3_C2Tx/CuS materials with high supercapacitor electrode applications [131]. Chen Q. et al. used the solvothermal method to prepare 3D porous nickel cobalt sulfide (NiCo_2_S_4_) electrodes for the supercapacitors application. The material achieved excellent energy storage performance due to its large specific surface area and high conductivity provided by the solvothermal process. The as-synthesis 3D-NiCo_2_S_4_ electrode presented a capacitance of 1211.6 F g^−1^ at 1 A g^−1^ and a stability retention at 80% after 3000 cycles [132]. A significant breakthrough in the specific capacitance of metal oxide supercapacitor materials was achieved by the hydrothermal process. This enhanced supercapacitor material was obtained by the two-step hydrothermal method constructed on nickel foam as the material substrate. The 3D porous electrode material synthesized by Ni-Co_2_O_4_@MnMoO_4_ demonstrated a high specific capacitance of 2603.9 F g^−1^ [133]. C. Bandas et al. developed Zn-ZnO(Nw)-rGO hybrid electrodes for supercapacitor applications, successfully prepared in situ by the microwave-assisted hydrothermal method. During the hydrothermal process, both deposition and reduction of graphene oxide were achieved on the structure of ZnO nanowires grown on Zn metal foil (previously obtained). Electrochemical characterizations revealed that the Zn-ZnO(Nw)-rGO structures function as negative electrodes, exhibit a non-ideal rectangular shape, and behave as a pseudo-capacitor. A maximum capacitance of 395.79 mF cm^−2^ was measured at a scan rate of 5 mV s^−1^ and a maximum specific capacitance of 145.59 mF cm^−2^ was determined at a low power density of 2 mA cm^−2^ [134]. Also, M.I. Morariu (Popescu) et al. conducted a study that focused on the growth of Cu_2_O/CuO nanowires by one-step thermal oxidation using a flexible copper mesh. The results of electrochemical measurements for the Cu/Cu_2_O/CuO(Nw) structure treated at 300 °C showed that it acts as a positive electrode, reaching the highest capacitance values of 26.158 mF cm^−2^ at a scan rate of 5 mV s^−1^, as well as a maximum specific capacitance of 21.198 mF cm^−2^ at a low power density of 0.5 mA cm^−2^ [135].

Electrochemical deposition is the process of applying electrochemical reactions to add layers of material on the surface of a substrate. This technique can be classified according to the defined potential as sub-monolayer deposition (SMD), which is generally the deposition of 2D structures and a single layer, while overvoltage deposition (OVD) is the deposition of 3D stacked structures. In OVD deposition, single as well as multiple three-dimensional nuclei can be formed so that a thin lattice or a thick lattice is grown on the substrate. The three-dimensional nucleation and growth of metals or alloys follows Volmer–Weber Island growth kinetics and can be controlled by the application of cathodic deposition overpotential. This overpotential determines the chemistry involved in forming the nuclei and the islands formed: conditionally tethered islands with asymmetric geometries, half-spheres in intermediate gas-liquid conditions, and spiral aggregates or dendritic structures [136]. High capacitance oxide materials electrodes were synthesized by a simple electro-deposition method. One of the most promising methods for enhancing the performance of supercapacitors is the electrodeposition of metal oxides on surfaces, particularly those with foam-like structures to increase surface area. However, from all the transition metal oxides, NiO is the most interesting material due to its high theoretical specific capacitance and strong redox activity. Recently, studies have been focused on binary metal oxides, such as NiCo_2_O_4_, which have even higher specific capacitance and better corrosion resistance and thus increase their electrochemical utility [137]. M. Song and his group successfully fabricated NiO/NiCo_2_O_4—_3D composites on Ni foam substrate using simple electrochemical deposition. By varying the time of deposition and other electrochemical parameters, the optimal conditions were established. A specific capacitor performance of the composite NiO/NiCo_2_O_4_/NF electrode was 717.8 mF cm^−2^ at 2 mA cm^−2^ with a capacitance retention of 74.8% [138].

**Figure 8:**
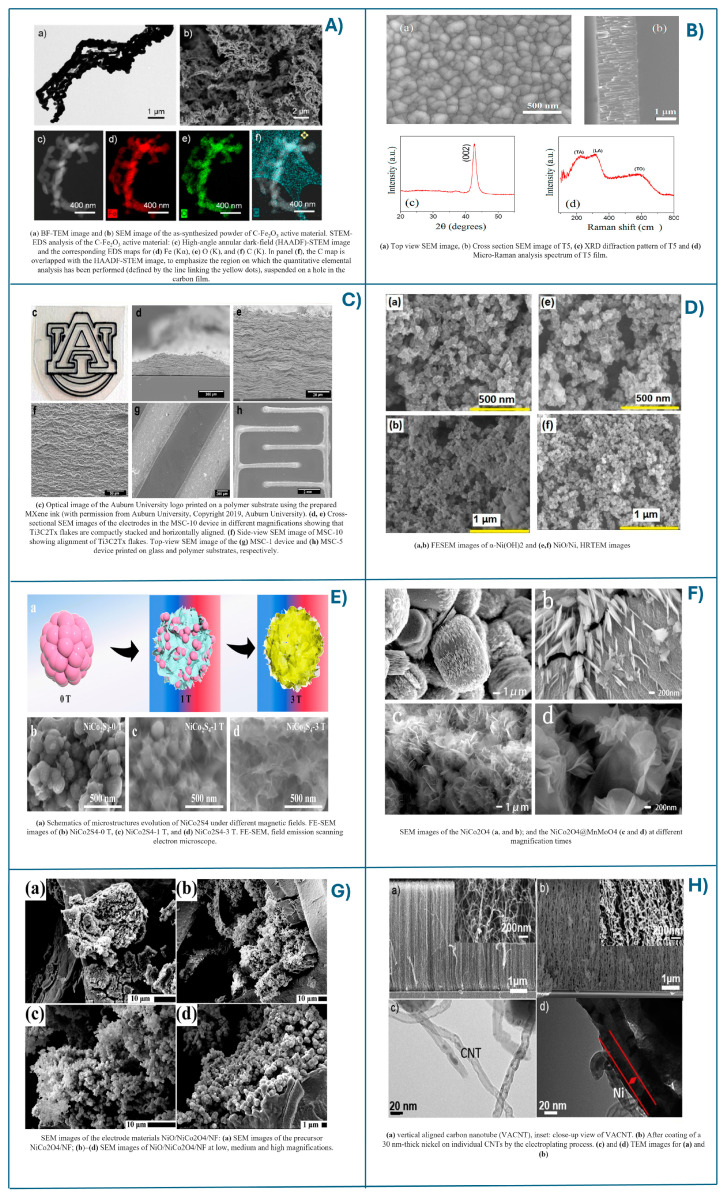
Different surface morphology and electrode fabrication for 2D and 3D supercapacitors from bottom-up approach: (**A**) Reprinted with permission from [119]. Copyright 2022, MDPI; (**B**) Reprinted with permission from [122]. Copyright 2015, Elsevier; (**C**) Reprinted with permission from [125]. Copyright 2020, American Chemical Society; (**D**) Reprinted with permission from [129]. Copyright 2022, MDPI; (**E**) Reprinted with permission from [132]. Copyright 2023, Elsevier; (**F**) Reprinted with permission from [133]. Copyright 2018, Elsevier; (**G**) Reprinted with permission from [138]. Copyright 2024, Elsevier; (**H**) Reprinted with permission from [143]. Copyright 2020, Elsevier.

Chemical vapor deposition (CVD) is a process where a gaseous chemical precursor hits the surface of a substrate and transforms into the desired solid material, which may exist as 2D thin films or 3D constructions. These 3D constructions can be realized by layering the thin 2D films or growing them in 3D forms constructed from foams, shells, and other complex designs. This widely used technique provides the capability of synthesizing materials, either in 2D or 3D, with controlled composition and structure [139,140]. CVD for supercapacitor applications initially focused on 2D materials like graphene. However, the practical capacitive performance of pure graphene often falls short, even in the case of a perfect layer [141]. In this way, other 2D graphene composites are of more interest, and Kumar S. and his group constructed an ultrahigh supercapacitor out of MXene covered with a nano graphite layer. It was observed that the capacitance of graphene-based supercapacitors was higher by more than 1.5 times than those in the absence of graphene. Cyclic voltammetry analysis revealed a high specific capacitance of about 542 F g^−1^ at a scan rate of 5 mV s^−1^ [142]. Zang X. and coworkers fabricated 3D graphene/CNT networks for hybrid supercapacitors using the CVD process. The combination of large surface areas (from graphene and CNTs), high electrochemical reaction sites (from graphene), and metal particles, such as pseudocapacitor materials, significantly improved the electrochemical capacitance. Compared to as-grown CNT forests, the capacitance of the 3D CNT/graphene networks increased 16 times to approximately 20 F cm^−3^, while the 3D CNT/CNT networks saw a 25-fold increase to approximately 30 F cm^−3^. A flexible supercapacitor based on the CNT/CNT forest electrode demonstrated a 90% retention of its original capacitance after 10,000 charge/discharge cycles [143]. By applying the bottom-up method for electrode fabrication with 2D and 3D structures for supercapacitors, different morphologies can be obtained, as shown in Figure 8.

Some synthesis methods, like the sol–gel method, also produce 2D and 3D material, after which the typical practice involves mixing these materials with an organic polymer and depositing them on a substrate using thin film deposition techniques: spin-coating, dip-coating, spray pyrolysis, or the Dr. Blade method. In the case of other methods, such as CVD, the desired material layers are created and deposited in a single step. While these techniques can be more costly than others that are available, they provide greater control over the layer thickness and crystallite size of the synthesized 2D and 3D materials. For example, methods such as 3D printing enable the easy formation of advanced 3D materials, but to produce the desired printed materials, a pre-printing processing step is first required.

### 4.2. The 2D Structures

Electrodes based on oxides offer higher energy density in contrast to carbon-based materials and better stability than conductive polymers. Nanostructures based on transition metal oxides such as Fe_2_O_3_, ZnO, NiO, CuO, TiO_2_, etc. are abundant in nature and nontoxic, providing enhanced thermal capacitance with excellent stability. These properties make them ideal materials for supercapacitor applications and can replace other electrode materials that have high energy density and powerful performance [144].

Layered 2D oxides are a new research class that has been studied in limited cases with no systematic record of development, promise, and opportunities for future work [97]. Lopa N. S. et al. developed a pseudocapacitor composed of electrodes of 2D SnO_2_-ZnO heterostructures on Au-modified SiO_2_/Si support. The electrode exhibited high energy storage capability and a capacitance retention of 96.3% after 5000 charge/discharge cycles [145]. In another study, Lopa N. S. et al. synthesized 2D SnO_2_-In_2_O_3_ heterostructures on Au/SiO_2_/Si wafers and demonstrated that the heterostructured electrodes have very good super-capacitive performance based on the electrochemical measurements. Thus, the specific capacitance (Cs) had a value of up to 1048.25 F g^−1^ and 757 F g^−1^ at a scan rate of 10 mV s^−1^ and a current density of 12 A g^−1^, as well as a great electrochemical stability with a retention of 96.15%, even after 5000 charge/discharge cycles [146]. Two-dimensional tin dioxide (SnO_2_) nanoplatelets/graphene nanocomposites were synthesized by Li Z. et al. and the electrochemical measurements showed that these electrodes have a very good specific capacitance of 294 F g^−1^ and excellent cycling stability, with a retention of 90%, even after 2000 charge/discharge cycles [147]. Adewinbi S.A. and coworkers have synthesized and characterized the TiO_2_ thin-film electrodes for optoelectronic and supercapacitor applications and deposited the thin films onto conductively coated indium tin oxide glass substrate using an electrochemical deposition technique. Therefore, adding 30 mL of 0.3 M TiSO_4_(aq) and 10 mL of 0.1 M NaOH(aq), the TiO_2_ thin films were grown from an electrolytic solution. The authors reported that the film electrodes had significant high-rate energy storage capacity and stability, representing an increase of the capacity approximately at 15.86 mF cm^−2^ and 9.19 mA h cm^−2^, respectively [148]. Kumar R.D. and team synthesized an ultra-stable TiO_2_ nanosphere for a high-performing supercapacitor electrode. At the current density of 0.5 A g^−1^, the maximum specific capacitance for the 2D TiO_2_ nanosphere-coated electrode was shown to be 810 F g^−1^ and the electrode exhibited a stability of almost 100% after 10,000 cycles. The supercapacitor had a maximum power density of 2313.2 W kg^−1^ and an energy density of 9.8 W h kg^−1^ [81]. For this reason, Nejkar T.M. et al. prepared a binder-free α-Fe_2_O_3_ thin film anode using the SILAR technique to fabricate a high-performance Mg-ion asymmetric supercapacitor and a two-dimensional α-Fe_2_O_3_ thin film electrodes with a capacitance of 381 F g^−1^ at 0.2 mA cm^−2^. The device assembled has demonstrated the ability to deliver a maximum specific capacitance of 234 F g^−1^, impressive energy density of 105.2 W h kg^−1^, and power density of 619.4 W kg^−1^. Moreover, one Mg-ISC device has shown excellent cyclic stability with 86% capacitance retention after 10,000 cycles [149]. In another study, Gund G.S. and team prepared MnO_2_ and Fe_2_O_3_ thin films directly on stainless steel substrate, and the authors grew MnO_2_ nanosheets on flexible and large-area stainless steel papers measuring 8 × 7 cm^2^ with potentiodynamic electro-deposition using an electrolytic solution of 0.1 M KMnO_4_ and 0.1 M KNO_3_, respectively. Fe_2_O_3_ nanoparticles were synthesized on pre-cleaned stainless-steel sheets through alternate immersion in iron sulfate and sodium hydroxide solutions at various temperatures (313, 333, and 353 K). A four-beaker SILAR system was employed, using 0.05 M FeSO_4_ and 0.1 M NaOH solutions as the cationic and anionic sources, respectively. The estimated values of specific capacitance for positive and negative electrodes are 333 F g^−1^ for MnO_2_ and 283 F g^−1^ for Fe_2_O_3_, respectively, at a 5 mV s^−1^ scan rate. Flexible solid-state supercapacitors (FSS-SCs) with MnO_2_ and Fe_2_O_3_ electrodes and a Na_2_SO_4_/CMC gel electrolyte were assembled. The asymmetric SC highlights a higher performance with a maximum specific capacitance of 92 F g^−1^, doubled energy density of 41.8 W h kg^−1^, excellent flexibility, and also good cycling stability [150]. Jacob O.B. and co-workers prepared Co_3_O_4_@Fe_2_O_3_-decorated 2DC via one-pot environmentally benign microwave combustion. Thus, the Co_3_O_4_@Fe_2_O_3_-2DC obtained showed an exciting specific capacitance of 2657.1 F g^−1^ at 1 A g^−1^ current density. The excellent electrochemical performance is due to the high surface area of the material, along with the synergistic interaction between the various components in the layer composite. Moreover, the electrode exhibited excellent cycling stability of about 96.7% capacitance retention after 4000 cycles at 5 A g^−1^ [151]. Akbari M. K. and his team utilized plasma nanoengineering treatment to deposit 2D Ga_2_O_3_ films in order to study supercapacitive behavior. Thus, 2D Ga_2_O_3_ films had a high specific capacitance of 113 F g^−1^ achieved at the current density of 2.6 A g^−1^. The capacitive retention of 87.7% was accomplished for 2D Ga_2_O_3_ materials after 1000 cyclic measurements. They observed that the capacitive retention decreases with thickness, so they used a thinner film of 35 nm for the next measurements [83]. Hu Y.-L. et al. studied the point defects effect of α-Ga_2_O_3_ for supercapacitor application. A 2D layer of α-Ga_2_O_3_ microrods was deposited on carbon cloth (CC) using a hydrothermal and post-annealing processes in the air. They emphasized that by maintaining the hydrothermal process over a 6 h period, the electronic conductivity was enhanced due to the reduction of the point defects number in α-Ga_2_O_3_, and the maximum capacitance was 1728 mF cm^−2^ at a current density of 1 mA cm^−2^. Also, the aqueous symmetrical supercapacitor Ga_2_O_3_/CC exhibited a specific areal capacitance of 1394 mF cm^−2^ at 0.5 mA cm^−2^ [62]. H. Xu et al. synthesized heterostructures based on 2D SnO_2_-Ga_2_O_3_ fabricated on a wafer by the atomic layer deposition technique. After annealing the n-p heterostructures at 250 °C for 1 h in the air, a high specific capacitance of 167 F g^−1^ at the current density of 7.69 A g^−1^ and a high capacitance retention (~92.55%) was obtained, even after 10,000 continuous cycles [60].

Besides the described metal oxide structures, other interesting compounds with good properties for supercapacitor application have been studied. Among these oxides, RuO_2_ with a high theoretical capacity is adequate for practical use in supercapacitors, but the main disadvantage is the high production cost and toxicity. High specific capacitance, good cyclic retention, reversible redox reactions with oxygen evolution reaction activity, and metallic conductivity were found in various studies for effective utilization applied as supercapacitor electrode material. In 2D supercapacitor applications, RuO_2_ is typically used via electrochemical approaches like electrodeposition [152]. U.M. Patil et al. studied the chemical synthesis of hydrous RuO_2_ thin films for use in supercapacitors. The films were suitably prepared at low temperatures onto glass and stainless-steel substrates, using a cost-effective chemical bath deposition (CBD) method. Cyclic voltammetry tests in a 0.5 M H_2_SO_4_ electrolyte showed that the hydrous RuO_2_ films produced a maximum specific capacitance of 73 F g^−1^ [153]. Kashif M. et al. developed a NiV_4_ electrode for supercapacitor application. Furthermore, the team investigated the interaction between 2D V_2_O_5_ nanosheets and Ni-based MOFs in material synthesis and functionality. This nanomaterial exhibited electrochemical applications such as water splitting and supercapacitors. The synthesized NiV_4_ electrode showed a specific capacitance of 546 F g^−1^ at a current density of 1 A g^−1^ for supercapacitor applications, with very slight capacitance loss after 10,000 charge/discharge cycles [154]. Mane et al. V.J. deposited 2D MnO_2_ thin films with a tetragonal and birnessite phase by the CBD and SILAR methods, respectively. 2D thin films exhibited excellent electrochemical performance with a maximum specific capacitance of 757 F g^−1^ at a scan rate of 5 mVs^−1^. When the devices were assembled into symmetric supercapacitors, their specific capacitance was 128 F g^−1^; energy density at a power density of 0.2 kW kg^−1^ was 14 W h kg^−1^ and maintained 90% of the capacitance after 5000 CV cycles [155]. S.A. Mane et al. prepared a flower-like Bi_2_O_3_ by rotation-al-chemical bath deposition (R-CBD) on flexible stainless-steel mesh substrates. In this way, the value of specific capacitance for Bi_2_O_3_ was 421.76 F g^−1^ at a current density of 10 mA, with an energy density of 149.95 W h kg^−1^ and a power density of 963.85 W kg^−1^. The electrode exhibited cycle stability of 60% after completion of 1000 cycles. This corresponds to an ASC device with a specific capacitance of Bi_2_O_3_||AC at 1 mA cm^−2^, while energy and power densities were 18.24 W h kg^−1^ and 1008.67 W kg^−1^, respectively, and the obtained retention was 83.67% after 1000 cycles at 2 mA cm^−2^ [156].

To improve the performance of supercapacitors, composite materials were intensively studied and used to achieve the electrodes. Thus, 2D carbon-based materials, like graphene, are well-known for their outstanding electrical properties and extensive surface area. For example, the theoretical specific capacitance of single-layer graphene is around 21 µF cm^−2^ and 550 F g^−1^ when fully utilized. However, the actual capacitive performance is often lower as a result of significant agglomeration during preparation and use. Therefore, improving the overall energy storage performance of graphene-based materials is still a major challenge [141]. To evidence the supercapacitor performance, the researchers used two main strategies: the functionalization of electrode materials and the development of composite electrodes, with some techniques merging both strategies. Composite electrodes are distinguished for their considerable advantages, such as improved electronic and ionic conductivities through synergistic effects, a greater surface area, and controlled morphologies. These attributes result in higher specific capacitance and energy density, taking full advantage of metal oxides [157]. Graphene-based materials have been extensively studied as a conductive network for supporting the redox reactions of transition metal oxides, hydroxides, and conducting polymers. These graphene/metal oxide composites, which combine graphene with nanoparticles of transition metal oxides or hydroxides, exhibit superior electrochemical performance. The graphene layers enhance the dispersion of metal oxide/hydroxide nanoparticles and serve as a highly conductive matrix, boosting electrical conductivity. Meanwhile, the metal oxides or hydroxides contribute pseudocapacitance, resulting in a synergistic effect that improves overall performance [158]. In a study, Amirabad T.N. et al. prepared a 2D-Zn@Fe_2_O_3_/MoS_2_@rGO nanocomposite through eco-friendly methods, which exhibited outstanding supercapacitor performance. A maximum of 3078 F g^−1^ at 1.0 A g^−1^ was achieved from the material. An asymmetric supercapacitor device, built using AC as a positive electrode, delivered an energy density of 38.88 W h kg^−1^ at a power density of 1000 W kg^−1^. The device exhibited excellent stability of about 90% capacity retention after 3000 cycles [159]. Qu Q. and coworkers demonstrated that the improved electrochemical performance of 2D sandwich-like Fe_3_O_4_-graphene nanocomposites results from maximizing the electrochemical surface area and achieving a synergistic effect in asymmetric capacitance. The Fe_3_O_4_ nanorods are formed by nucleating FeOOH nanorods on the oxygen groups of graphene oxide (GO) and then undergoing electrochemical cycling. This hybrid composite exhibited a specific capacitance of 304 F g^−1^—2.5 times higher than comparable materials—and maintained capacitance retention over 1000 cycles in supercapacitor devices. Additionally, Fe_3_O_4_-graphene nanocomposites provided higher power density, despite their modest capacitance, due to their negative working potential, outperforming other high-capacitance electrodes such as nickel and cobalt oxides [160]. In another study, Iqbal M. et al. obtained a high-performance supercapacitor based on 2D-MoS_2_@TiO_2_ composite electrodes by a simple hydrothermal method. The MoS_2_@15%TiO_2_ binary composite presented a maximum specific capacitance of 210 F g^−1^ at 10 mV s^−1^ with a capacitance retention of 98% over 2000 cycles [161].

Therefore, graphene-based composite materials have been intensively used for obtaining the electrodes for supercapacitors due to their specific characteristics. Yan J. and team developed graphene–MnO_2_ composites for supercapacitors, achieving a high specific capacitance of 310 F g^−1^ at 2 mV s^−1^ and 228 F g^−1^ at 500 mV s^−1^, nearly three times greater than pure graphene and birnessite-type MnO_2_. This composite demonstrated exceptional performance, retaining 95.4% of its capacity after 15,000 cycles at 500 mV s^−1^. The established performance is attributed to the well-dispersed MnO_2_ nanoparticles of small size (5–10 nm) on graphene, which increase ion buffering, fast electron transfer, and interfacial contact, thereby reducing voltage drop and stability [162]. Another structure based on NiO nanoflakes/graphene nanocomposites was developed by Zhu et al., for supercapacitor electrodes using a controlled hydrothermal method. Following the hydrothermal process, the NiO nanoflakes were uniformly attached to the graphene sheets, forming a metal oxide composite material. Also, the enhancement of this composite is due to the construction of efficient diffusion channels for both electronic and ion transport, which helped to prevent the aggregation of the NiO nanoflakes. As a result, they have achieved significantly higher specific capacitance values such as 240 F g^−1^ at 5 A g^−1^ and 220 F g^−1^ at 10 A g^−1^, in contrast to neat NiO electrodes that only reached 100 F g^−1^ at 5 A g^−1^. Additionally, the NiO nanoflake/graphene nanocomposites showed remarkable cycling stability, maintaining 100–120% capacity retention after 1500 cycles [163]. Xiong D. and colleagues developed free-standing, flexible Co_3_O_4_/graphene foam (GF) hybrid films using a calcination process. The high mass loading of Co_3_O_4_ combined with the synergistic effects of the electrochemically active Co_3_O_4_ and the conductive GF allowed the Co_3_O_4_/GF electrodes to exhibit exceptional electrochemical performance without the need for current collectors or binders. The Co_3_O_4_ phase in the hybrid achieved a high capacitance of 652 F g^−1^ after 3000 cycles at a current density of 2 A g^−1^, which is four times higher than that of pristine Co_3_O_4_. Additionally, a superior rate capability of 665 F g^−1^ was observed at a high current density of 20 A g^−1^. These results highlight the significant potential of these flexible hybrid film electrodes for use in wearable energy storage devices [164].

Pradeepa S.S. et al. reported 2D layered MXene of Ti_3_C2 decorated with ZrO_2_ nanospheres through the sonication method. The nano-composite electrode Ti_3_C2-ZrO_2_ obtained showed excellent performances, a specific capacity of 483.6 C g^−1^ at 1 A g^−1^, and capacitance retention of 88.85% after 5000 cycles. The Ti_3_C2-ZrO_2_//AC solid-state hybrid supercapacitor device has delivered an energy density of 75.60 W h kg^−1^ and a power density of 1445 W kg^−1^ [165].

### 4.3. The 3D Structures

Many studies have been performed to improve the energy density of supercapacitors by optimizing the design of the 3D nanostructured metal oxide electrodes and/or by using 3D nanoporous current collectors. These designs ensure large surface areas and open pores, improving ionic and electronic transfer. The result was an increase in the energy density due to a higher mass loading of active materials. Further, the additional faradic capacitance from in situ surface oxidation of the current collectors is an important factor for the utilization of metal oxide-like supercapacitor materials [76]. W. Tian et al. developed a 3D hybrid electrode material using a simple and scalable two-step process. Firstly, α-Fe_2_O_3_ nanowires were synthesized by oxidizing a pure iron foil in the air at 600 °C for 5 h and the layer that formed was collected after cooling. Afterward, core/shell hybrid samples were obtained by immersing the Fe foil with Fe_2_O_3_ nanowires into a 0.1 M Ni(NO_3_)_2_ solution and treating them at 85 °C for 5 h to grow Ni(OH)_2_ nanosheets. In terms of their performance, after 5000 charge/discharge cycles, the hybrid electrode maintained a specific capacitance of 390 F g^−1^, retaining 85.7% of its initial value, which is significantly better than the Ni(OH)_2_ nanosheet electrode, which had a capacitance of 247 F g^−1^ with a retention of 79.7% [166]. Meena A. et al. prepared a hierarchical Fe_2_O_3_ nanosheet attached to CoMn-layered double hydroxide nanowire as a 3D electrode with high capacitance, which enhanced the limitations of single-component metal oxides. It was fabricated by a multistep hydrothermal process to form a 3D electrode with doubled hydroxide nanowires anchored onto NF. Hydrothermal growing Fe_2_O_3_ nanosheets were performed on CoMn-layered double hydroxide nanowires. The resulting hybrid electrode exhibited a high specific capacitance of 2633 F g^−1^ at 1 A g^−1^ and maintained a coulombic efficiency of approximately 99% after 5000 cycles [167]. Guo W. et al., grew a 3D vertically aligned TiO_2_ nanowire array film on a titanium substrate via a two-step combining method that involves a chemical oxidation process with melamine and a subsequent pyrolysis process. The TiO_2_ NWA electrode was assembled in an asymmetric configuration with Ni foam/porous carbon (NFPC) as the positive electrode. The assembled asymmetric supercapacitor NFPC//Ti-TiO_2_ achieved a high cell voltage of 2.3 V and a volumetric energy density of 6.27 mWh cm^−3^ at a power density of 63.4 mW cm^−3^ [168]. In another study, A. Ali et al. synthesized a 3D core-shell structure of NiO(NW)@NiO nanosheets grown on nickel foam, and the resulting electrode presented an excellent electrochemical performance. This approach was managed to obtain a high specific capacitance of 1782 F g^−1^ at 1 A g^−1^. In addition, good stability was maintained at 70.33% of its initial capacitance after 7000 galvanic charge/discharge cycles [169]. Other researchers have produced some NiO microflowers that have an impressive supercapacitor performance, with a specific capacitance of 1828 F g^−1^ at 0.5 A g^−1^. Additionally, a hierarchical porous array of NiO nanotubes was developed and characterized for supercapacitor applications. This assembly exhibiting in a symmetrical supercapacitor configuration the maximum specific capacitance of 675 F g^−1^ at a current density of 2 A g^−1^ was achieved [170].

Li Y. et al. prepared nanoporous copper (NPC) by cleaving ZrCuAl metallic glass ribbons with HF acid solutions, yielding tunable ligament sizes ranging from 20 to 300 nm. They created a nanoporous composite by oxidizing NPC with ethanol to form NPC/Cu_2_O. The NPC/Cu_2_O composite exhibited a specific capacitance of 190.93 F g^−1^ at a scan rate of 0.005 Vs^−1^. From this study, the nanoporous composite electrode demonstrated good electrochemical performance and showed significant potential for use in flexible and high-performance energy storage devices [171]. Sun X. and Jian Z. prepared a 3D net-like Co_3_O_4_@NiO nanostructure by a two-step hydrothermal process with subsequent annealing, supported on Ni foam. The obtained 3D network had a large specific surface area, which contributed to an enhanced electrochemical performance. It showed that the composite electrode had a specific capacitance of 1306 F g^−1^ at a current density of 1 A g^−1^ and excellent cycling stability, with a capacitance retention of 95.5% after 3000 cycles, even at a higher current density of 8 A g^−1^ [172]. Ge W. et al. demonstrated that the dealloying process is an efficient way to fabricate nanoporous nickel (NPN) with tunable pore sizes. Through a subsequent conversion process on the surface using various chemical solutions, they synthesized Ni(OH)_2_ nanosheets on the NPN surface and obtained highly promising results, the Ni(OH)_2_@NPN electrode exhibited an ultrahigh specific capacitance of 3790 F g^−1^ at 2 A g^−1^ and excellent capacitance retention of 92% after 5000 cycles. These results indicate that the strategy of combining dealloying with surface conversion is appropriate for high-performance supercapacitor electrodes [173]. Chen P.C. et al. achieved a 3D network using an electrochemical dealloying process on a commercial “Nitinol” alloy in nitric acid electrolyte, followed by annealing at 450 °C for 30 min, presenting a capacitance of 35.5 F g^−1^ at 20 mVs^−1^ [174].

The hybrid structures based on graphene compound for electrode development were studied to enhance the supercapacitor performance. The 3D graphene-based hybrid electrodes show a promising energy storage potential due to the mass loading of active materials in these electrodes remaining low. Increasing the amount of active pseudocapacitive materials in the 3D hybrid electrode structure was demonstrated by many research groups to be crucial for future advancements in supercapacitors [175]. In 3D metal oxide supercapacitors, the charge storage mechanism is based on electrochemical faradaic processes. During this oxidation/reduction reaction, many free electrons move across the electrode–electrolyte interface. The advantage is that these electrons do not create additional chemical species, achieving greater stability and longer life of the electrode materials. The structure and particle size distribution of the 3D metal oxide materials are key factors contributing to their pseudo-capacitance. These metal oxides serve as the core materials in such pseudo-capacitors [176]. Zamiri G. et al. have developed an ASC using the ternary nanocomposite of three-dimensional graphene−tin oxide−titanium dioxide (3DG−SnO_2_−TiO_2_), and the assembled device showed a specific energy of 28.6 Wh kg^−1^ and a specific power energy of 367.7 W kg^−1^ at 0.5 A g^−1^ current density. Moreover, ASC demonstrated excellent cycling stability and maintained 97% of its original capacity after 5000 cycles [177]. Ke Q. et al. have successfully achieved a new 3D nanostructure containing ultrathin Ni(OH)_2_ nanoflakes, anchored directly on SnO_2_ nanowire surface arrays. SnO_2_/Ni(OH)_2_ (core-shell) nanocomposites exhibited a specific capacity of up to 1553 F g^−1^ at 0.5 A g^−1^ and at a high value of current density of 10 A g^−1^, the electrode maintained a high capacitance value of 934 F g^−1^ [178]. In another study, Zhang et al. produced and used positive and negative electrodes to develop an asymmetric supercapacitor made of graphene oxide/carbon cloth (rGO/CC) and 3D lamellar SnO_2_ grown on a CC substrate. The obtained device showed a very good cycle stability of 76.9% after 10,000 cycles at 3 A g^−1^ and a high energy density of 22.8 W h kg^−1^ at a power density of 850 W kg^−1^ under a cell voltage of 1.7 V [179]. Narsimulu D. et al. have developed a 3D porous SnO_2_ on conductive carbon cloth composite utilized as an anode for lithium-ion batteries (LIBs) and sodium-ion batteries. Experiments have shown that the 3D porous SnO_2_/CC composite electrode exhibits excellent electrochemical properties for both lithium and sodium storage. Thus, this structure can be used successfully in the achievement of supercapacitors and more [180]. An enhancement of 3D Fe_2_O_3_ electrodes by incorporating unique structures, such as Fe_2_O_3_/GNs/CNTs, was demonstrated by Tian Y. and team. This electrode exhibited a significantly improved specific capacitance of 675.7 F g^−1^ at 1 A g^−1^, surpassing the performance of Fe_2_O_3_ (322.6 F g^−1^) and Fe_2_O_3_/GNs (606.5 F g^−1^), as well as previously reported Fe-based electrodes [181]. The team, coordinated by Peiwen Ju, synthesized a 3D TiO_2_/RGO/MoO_2_@Mo electrode with an appropriately well-designed architecture through a one-step hydrothermal process followed by an in-situ growth route on a Mo net. The as-obtained electrode exhibited a real capacitance of 3927 mF cm^−2^ at 3 mA cm^−2^ and 96.5% capacitance retention after 5000 cycles. That would be due to amorphous TiO_2_ and MoO_2_ particles offering plenty of active sites and accommodating volume expansion, while MoO_2_ nanorods and walnut-shaped spheres enhance electron transport. RGO serves as a flexible scaffold to buffer the volume changes in the charge/discharge process [182].

Li Y. et al. proposed a 3-D N-doped Ti_3_C2/TiO_2_ hollow sphere electrode by using a self-sacrificing template method. The as-obtained electrode exhibited an impressive specific capacitance of 572 F g^−1^ at 1 A g^−1^ with excellent cyclic stability of 99.6% capacitance retention after 9500 cycles. The symmetric supercapacitor assembled showed a capacitance of 157 F g^−1^ at 1 A g^−1^, with a remarkable energy density of 17.7 W h kg^−1^ at the power density of 450 W kg^−1^ [183]. Li J. et al. obtained a 3D nano-branched TiO_2_-carbon nanotube by electrochemical deposition of TiO_2_ nanostructures on the CNT film. The maximum capacitance of the electrode was 283 F g^−1^ at 25 mV s^−1^. Also, the symmetric solid-state supercapacitor’s device presented a specific capacitance of 345.7 F g^−1^ at 1.0 A g^−1^ and the 3D composite electrodes show excellent cyclic stability with a retention of 93.3%, after 10,000 cycles [184]. Hu Y.-L. et al. synthesized 3D composite structures by hydrothermal growth of GaN/Ga_2_O_3_ on carbon cloth, which was used as a high-performance electrode for supercapacitors. Thus, a symmetric setup was assembled using both 1 M H_2_SO_4_ aqueous solution and PVA-H_2_SO_4_ gel as electrolytes. GaN/Ga_2_O_3_/CC-500 (annealing temperature of 500 °C) symmetric aqueous supercapacitor exhibited a high capacitance of 1301.20 mF cm^−2^ at 0.5 mA cm^−2^. The solid electrolyte version demonstrated good performance, reaching a capacitance of 1183.35 mF cm^−2^. After 20,000 cycles at 10 mA cm^−2^, the aqueous supercapacitor showed a capacity retention of 75.23% and a solid state of 77.27% of the initial value [61].

Therefore, recently, some studies have been made to develop 3D structures based on other oxides besides those previously described. In this way, Liu S. and colleagues optimized a 3D interconnected foam-like NiO@rGO composite as a binder-free electrode, where the advantages of combining transition metal oxides with highly conductive carbon materials were very clearly presented. The NiO@rGO-250 composite demonstrated a specific capacitance of 1399 F g^−1^ at 1 A g^−1^, and superior cycling stability compared to pristine materials. Additionally, an asymmetric supercapacitor assembled using NiO@rGO and activated carbon electrodes achieved a high specific energy of 40.4 W h kg^−1^ and a specific power of 750 W kg^−1^ [185]. In another study, Prabhu S. et al. developed a novel 3D flower-like CuO/Co_3_O_4_/r-GO heterostructure using a hydrothermal synthesis method for high-performance supercapacitor anodes. This CuO/Co_3_O_4_/r-GO heterostructure presented an impressive specific capacitance of 1458 F g^−1^ at a current density of 0.5 A g^−1^. This exceptional rate capability and stable long-term cycling performance, at 97% of its capacitance after 10,000 charge/discharge cycles at 5 A g^−1^, make this composite a promising material for supercapacitors [186]. Abaft E. et al. synthesized a 3D GO(Aerogel)/NiFe_2_O_4_ composite with a high surface for energy storage applications. The hybrid supercapacitor was fabricated based on the processing of GO(Aerogel) and NiFe_2_O_4_ via a hydrothermal procedure, followed by freeze-drying at −30 °C for 24 h. GO(Aerogel)/NiFe_2_O_4_ nanocomposite exhibited a maximum specific capacitance of 1393 F g^−1^ at 1 A g^−1^, along with retaining 82% of its capacitance after 1000 cycles. In this asymmetric assembled configuration, a high energy density of about 61.73 W h kg^−1^ and a power density of 570.25 W kg^−1^ were revealed, showing its potential in the application of supercapacitors for energy storage [187]. Li Y. et al. developed a 3D hierarchical NiCo_2_O_4_ structure grown on few-layered Ti_3_C_2_ MXene deposited on NF as substrate material, showing a high specific capacitance of 2468 F g^−1^ at a current density of 0.5 A g^−1^ due to the synergistic effects of the materials used. Also, they further assembled an asymmetric supercapacitor using NiCo_2_O_4_/Ti_3_C_2_ as the positive electrode and AC/NF as the negative electrode. The device displayed the highest capacitance of 253 F g^−1^ at 1 A g^−1^ and showed 91.5% capacitance retention after 10,000 cycles [188].

## 5. Conclusions

Because of the diversity of applications in different crucial domains for humanity, the need for devices with energy storage is constantly growing. Obviously, research into the production of electrode materials used in electrochemical energy storage systems is critical, and more investments are being directed toward this area. Electrochemical supercapacitors are emerging as essential energy storage devices due to their high-power density and long cycle stability. Among all the types of existing materials, metal oxides are the most promising for making electrodes because, as demonstrated, the contact surface of the electrode with the electrolyte is greatly improved, resulting in the facilitation of electrochemical redox reactions, ultimately leading to the spectacular improvement in supercapacitor performance. The use of innovative electrodes based on nanostructured metal oxides allows the improvement of very important parameters for the optimization of supercapacitors and their adaptability for specific applications. The 2D and 3D nanostructures have significantly transformed supercapacitors, bringing about a great improvement in energy storage capabilities. The incorporation of 2D and 3D structures of metal oxides and other classes of materials increases the surface area, conductivity, and overall electrochemical performance, such as the high theoretical specific capacitance of supercapacitor electrodes, thereby significantly improving their efficiency and effectiveness. The formation of pores and the building of hierarchical structures in both 2D and 3D oxide nanostructures can significantly increase electrode/electrolyte contact area, reduce ion diffusion distances, improve structural stability, and provide additional active sites. In addition, a very common method to achieve better electrical properties is the functionalization of these oxides with some conductive materials to form heterostructures used in obtaining the electrodes for supercapacitors. 3D supercapacitors made with metal oxide active materials present energy densities greater than conventional batteries if the active material content in the nano-porous electrodes is higher than 50%. It all depends on the structure of these metal oxides, the design of the nanoporous electrodes, and the way they interact with current collectors and electrolytes. Thus, special attention should be addressed to the research aimed at fine-tuning these variables in order to create the future generation of 3D supercapacitors with higher energy densities.

## Data Availability

Not applicable.
